# Peroxisomes Implicated in the Biosynthesis of Siderophores and Biotin, Cell Wall Integrity, Autophagy, and Response to Hydrogen Peroxide in the Citrus Pathogenic Fungus *Alternaria alternata*

**DOI:** 10.3389/fmicb.2021.645792

**Published:** 2021-06-28

**Authors:** Pei-Ching Wu, Yu-Kun Chen, Jonar I. Yago, Kuang-Ren Chung

**Affiliations:** ^1^Department of Plant Pathology, College of Agriculture and Natural Resources, National Chung Hsing University, Taichung, Taiwan; ^2^Plant Science Department, College of Agriculture, Nueva Vizcaya State University, Bayombong, Philippines

**Keywords:** autophagy, MAP kinase, peroxisome, siderophore, singlet oxygen, virulence

## Abstract

Little is known about the roles of peroxisomes in the necrotrophic fungal plant pathogens. In the present study, a *Pex6* gene encoding an ATPase-associated protein was characterized by analysis of functional mutations in the tangerine pathotype of *Alternaria alternata*, which produces a host-selective toxin. Peroxisomes were observed in fungal cells by expressing a mCherry fluorescent protein tagging with conserved tripeptides serine-lysing-leucine and transmission electron microscopy. The results indicated that Pex6 plays no roles in peroxisomal biogenesis but impacts protein import into peroxisomes. The number of peroxisomes was affected by nutritional conditions and H_2_O_2_, and their degradation was mediated by an autophagy-related machinery termed pexophagy. Pex6 was shown to be required for the formation of Woronin bodies, the biosynthesis of biotin, siderophores, and toxin, the uptake and accumulation of H_2_O_2_, growth, and virulence, as well as the Slt2 MAP kinase-mediated maintenance of cell wall integrity. Adding biotin, oleate, and iron in combination fully restored the growth of the *pex6*-deficient mutant (Δ*pex6*), but failed to restore Δ*pex6* virulence to citrus. Adding purified toxin could only partially restore Δ*pex6* virulence even in the presence of biotin, oleate, and iron. Sensitivity assays revealed that Pex6 plays no roles in resistance to H_2_O_2_ and superoxide, but plays a negative role in resistance to 2-chloro-5-hydroxypyridine (a hydroxyl radical-generating compound), eosin Y and rose Bengal (singlet oxygen-generating compounds), and 2,3,5-triiodobenzoic acid (an auxin transport inhibitor). The diverse functions of Pex6 underscore the importance of peroxisomes in physiology, pathogenesis, and development in *A. alternata*.

## Introduction

Peroxisomes are membrane-enclosed organelles found in all eukaryotic cells. Although fatty acid metabolism occurs in both peroxisomes and mitochondria in mammalian cells, peroxisomes are the primary locations of fatty acid β-oxidation in fungi and plants (Wanders et al., 2010; Titorenko and Rachubinski, 2011). Breakdown of fatty acid in peroxisomes leads to the accumulation of high levels of H_2_O_2_ and other highly toxic reactive oxygen species (ROS), including superoxide and hydroxyl radicals. To alleviate the toxicity of ROS, peroxisomes also contain a number of antioxidant enzymes. In addition to fatty acid β-oxidation and H_2_O_2_ detoxification, peroxisomes play a wide range of metabolic, physiological, and developmental functions in different organisms. In humans, peroxisomes play a key role in the synthesis of cholesterol, bile acids, and plasmalogens, and defects in peroxisome biogenesis could cause severely neurological disorders (Gould and Valle, 2000). In plants, peroxisomes are involved in the biosynthesis of phytohormones, biotin, and secondary metabolites (Hu et al., 2012; Maruyama et al., 2012). Peroxisomes are also involved in plant immune response (Lipka et al., 2005). In fungi, peroxisomes are involved in the biosynthesis of secondary metabolites (Bartoszewska et al., 2011; Stehlik et al., 2014), glucose catabolism (Idnurm et al., 2007), appressorium formation, and virulence (Kimura et al., 2001; Wang et al., 2007; Fujihara et al., 2010; Min et al., 2012).

Peroxisomes are dynamic organelles whose biogenesis and degradation are intricately regulated in order to adapt to the changing environment (Platta and Erdmann, 2007a; Smith and Aitchison, 2009). It has long been accepted that *de novo* biogenesis of peroxisomes is originated by budding from endoplasmic reticulum (Tabak et al., 2008; Mahalingam et al., 2021). The number of peroxisomes could be increased by division of pre-existing ones as well (Titorenko and Rachubinski, 1998; Holroyd and Erdmann, 2001; Perry et al., 2009). Excess peroxisomes are degraded through pexophagy, an autophagy-related machinery (Sakai et al., 2006; Oku and Sakai, 2010; Nuttall et al., 2014; Law et al., 2017; Farré et al., 2019). Formation and maintenance of peroxisomes are surprisingly complex. More than 32 peroxin (Pex) proteins have been identified to be required for peroxisome biogenesis and protein import into peroxisomes (Platta and Erdmann, 2007a; Smith and Aitchison, 2009; Farré and Subramani, 2016). Most of the proteins targeting to peroxisomes contain a *p*eroxisomal *t*argeting *s*ignal 1 (PTS1) with conserved tripeptides serine-lysing-leucine (SKL) at their carboxyl terminus, which can be recognized by the import receptor Pex5 (Heiland and Erdmann, 2005; Brocard and Hartig, 2006). Pex5 contains six tetratricopeptide repeats (TPRs) at its C terminus, which can recognize and bind to the PTS1-containing proteins. Pex5-matrix protein complex attaches to the peroxisomal membrane *via* two docking proteins Pex13 and Pex14. The matrix protein is eventually released into peroxisomes *via* a cargo translocation and release machinery involving several peroxisome membrane proteins (e.g., Pex2, Pex8, Pex10 and Pex12; Platta and Erdmann, 2007b). After the matrix protein is released into peroxisomes, Pex5 can be recycled back to the cytosol *via* the ubiquitin-conjugating enzyme Pex4 and its accessory protein Pex22 (Dammai and Subramani, 2001; Gould and Collins, 2002). Although peroxisomal matrix protein translocation does not require energy, both the disassociation of Pex5 from the matrix protein and the ubiquitination process require ATP. The required energy is provided by two peroxins, Pex1 and Pex6, belonging to the members of the ATPase-associated (AAA) protein family (Rucktäschel et al., 2011). Pex1 physically interacts with Pex6 and together partially attached to the peroxisomal membrane with the assistance of Pex15.

Although peroxisomes are ubiquitously present in all eukaryotes, their functions could vary considerably between and within species. For example, Pex6-mediated protein import to peroxisomes has very different functions in two closely related *Alternaria alternata* pathotypes, which are mainly different in host plant preference. Deletion of the *Pex6* gene homolog in the Japanese pear pathotype of *A. alternata* slightly reduces vegetative growth and conidiation, and completely shuts down the production of the host-selective AK toxin (Imazaki et al., 2010). By contrast, deletion of the *Pex6* homolog in the tangerine pathotype of *A. alternata*, which produces a host-selective toxin named *Alternaria citri* toxin (ACT), results in a severe growth reduction, causes a moderate reduction in toxin production, but has no effects on conidiation (Wu et al., 2020a). The *pex6*-deficient mutant of the tangerine pathotype also reduces the formation of an appressorium-like structure (small, nonmelanized enlargement of hyphal tips), cell viability, and pathogenicity. The abilities to produce the host-selective toxin, acquire iron, degrade host cell wall, and detoxify ROS all have profound impacts on the tangerine pathotype pathogenesis. Many antioxidants are responsible for ROS detoxification, which are regulated by the Yap1 bZip transcription regulator (Lin et al., 2009, 2011; Yang et al., 2009), the Skn7 response regulator (Chen et al., 2012), the Hog1 mitogen-activated protein kinase (MAPK; Lin and Chung, 2010; Chung, 2013; Yu et al., 2016), and the Tfb5 basal transcription factor II (Fu et al., 2020) in the tangerine pathotype of *A. alternata*. ROS detoxification is also impacted by the NADPH oxidases (Yang and Chung, 2012, 2013), the major facilitator superfamily (MFS) transporters (Chen et al., 2017; Lin et al., 2018), and the siderophore-mediated iron uptake (Chen et al., 2013, 2014; Chung et al., 2020), as well as the glutaredoxin and thioredoxin systems (Yang et al., 2016; Ma et al., 2018). Because peroxisomes contain high levels of catalase (Schrader and Fahimi, 2006; Antonenkov et al., 2010), we reasoned that peroxisomal functions might play a role in cellular resistance to ROS in the tangerine pathotype. However, mutational inactivation of *Pex6* in this pathotype has no effects on ROS sensitivity (Wu et al., 2020a).

In this study, we investigated the functions of Pex6 in the biosynthesis of siderophores and biotin, cell wall integrity, formation of Woronin bodies, peroxisome dynamics in response to hydrogen peroxide, and pathogenicity in the tangerine pathotype of *A. alternata*. The results revealed that deletion of a single *Pex6* gene has drastic effects on all above-mentioned phenotypes, indicating an extraordinary role of the Pex6-mediated protein import into peroxisomes and perhaps peroxisome themselves in developmental, physiological, and pathological functions in fungi.

## Materials and Methods

### Fungal Strains and Culture Conditions

The wild-type strain of *A. alternata* (Fr.) Keissler used as a recipient host for transformation and mutagenesis experiments was obtained from a diseased citrus leaf and has been previously characterized (Lin et al., 2009). Two *pex6* mutant (Δ*pex6*) strains M4 and M9 were independently identified by targeted deletion of the *Pex6* gene in wild-type (Wu et al., 2020a). The CP4 strain was identified by transforming and expressing a functional copy of *Pex6* in Δ*pex6*. Two *slt2* deficient mutants (Δ*slt2*-D1, D2) and the CP6 complementation strain were identified from separate studies (Yago et al., 2011). Fungal strains were grown on potato dextrose agar (PDA, Difco, Sparks, MD, United States) or broth (PDB), or minimal medium (MM; Chung and Lee, 2014) at 28°C under constant fluorescent light or in complete darkness for 3–5 days. Conidia were collected from the surface of fungal colony with sterile water. Fungal transformants were recovered from a regeneration medium (RMM) containing sucrose as an osmotic agent and 5 μg/ml of sulfonylurea (Chem Service, West Chester, PA, United States) as in Chung and Lee (2014). For medium shift experiments, fungal mycelium grown in PDB for 2 days was collected by passing through a sterile paper filter, washed, transferred into MM amended with or without FeCl_3_, and incubated for additional 24 h.

### Sensitivity Tests

Sensitivity tests were conducted on PDA or MM containing a test compound: oleic acid (0.1 and 0.5%, dissolved in 95% ethanol), biotin (0.5 and 5 μM, dissolved in 10% DMSO), 2,3,5-triiodobenzoic acid (0.2–0.5 mM, dissolved in ethanol), FeCl_3_ (0.2 mM), eosin Y (50 μM), rose Bengal (15 μM), or 2-chloro-5-hydroxypyridine (1.5 mM). Fungal growth under constant fluorescent light of intensity 40 μE m^−2^ s^−1^ or in complete darkness was measured at 3–7 days. Each treatment contained three replicates, and experiments were repeated at least three times. Growth inhibition rates were calculated by dividing the comparative difference of the growth by the wild-type growth and multiplying by 100.

### Molecular and Genetic Procedures

A mCherry or mCherry-SKL-coding gene fragment was amplified by PCR from a pmCherry vector (Takara Bio, Shiga, Japan) with a forward primer mCherry-F-new pairing with a reverse primer mCherry-R or mCherry-SKL-R (Supplementary Table S1). Amplicons were independently fused with the *trpC* promoter and the terminator by the two-step fusion PCR approach. The resulting fragments [*trpC* promoter/mCherry (or mCherry-SKL)/*trpC* terminator] were independently cloned into a pCB1532 plasmid carrying the *Magnaporthe grisea* acetolactate synthase gene cassette conferring sulfonylurea resistance (Sweigard et al., 1997). Oligonucleotide recognition sites, BamHI and HindIII, were incorporated into the 5' and 3' primers, respectively, to facilitate cloning. Plasmid constructs were propagated in *Escherichia coli* DH5α and purified using a plasmid purification kit (BioKit, Taipei, Taiwan). Fungal protoplasts were prepared by incubating fungal hyphae with cell wall-degrading enzymes (CWDEs) in an osmotic solution as previously described (Chung et al., 2002). Plasmid constructs were introduced into fungal protoplasts using CaCl_2_ and polyethylene glycol (PEG 3350)-mediated transformation. RNA was isolated from fungal mycelium using TRI reagent (Sigma-Aldrich, St. Louis, MO, United States) followed by PureLink RNA Mini kit (Invitrogen, Waltham, MA, United States), treated with RQ1 RNase-free DNase (Promega, Madison, WI, United States), and used to synthesize cDNA using a One Step iScript cDNA synthesis kit (Bio-Rad, Hercules, CA, United States) and an oligo-dT primer. Real-time PCR reactions were set up using iQ SYBR Green Supermix (Bio-Rad) and performed in a CFX Connect model of Real-Time PCR Detection System (Bio-Rad). Amplification of the *A. alternata β*-tubulin gene was used as an internal control to normalize for variation among samples. The relative expression levels were calculated using a comparative CT method (ΔΔCT method; Schefe et al., 2006).

### Purification and Assays of Siderophores, Chitins, Melanin, and Toxin

The production of siderophores was assessed by growing fungal strains on medium containing chromeazurol S (CAS), hexadecyltrimethyl ammonium bromide (HDTMA), and FeCl_3_ (Schwyn and Neilands, 1987), which formed a blue complex. Removing iron from the complex by siderophores led to the formation of an orange halo around the fungal colony in which the bigger halo was indicative of the greater production of siderophores. For purification of siderophores, fungal strains were grown in PDB for 2 days, shifted to liquid MM, and incubated for additional 5 days. Siderophores were purified from culture filtrates of fungal strains by passing through a column pre-packed with Amberlite XAD-16 resins (Sigma-Aldrich) and eluting with methanol. After methanol was air-dried, siderophores were dissolved in 1 ml methanol and analyzed by thin-layer chromatography (TLC) as previously described (Chen et al., 2013). After methanol was evaporated, samples were dissolved in 0.2% formic acid in Milli-Q water and analyzed by high-performance liquid chromatography (HPLC). Chromatographic separation was carried out at room temperature with Ascentis C-18 column (5 μm particle size silica, 4.6 i.d. x 250 mm) by Agilent 1100 Series HPLC Value System (Santa Clara, CA, United States). The mobile phase consisted of a linear gradient of 0.2% formic acid in water and 0.2% formic acid in acetonitrile (from 75:25 to 25:75 in 15 min and 0:100 for last 5 min) and a flow rate of 1.0 ml/min. Siderophores were detected using a diode array detector at 220 nm. The identity of siderophores was validated by liquid chromatography/tandem mass spectrometry (LC–MS/MS).

Fungal chitin was purified from fungal mycelium by heating in 6 N HCl and quantified by measuring the acid-released glucosamine from chitin using *p*-dimethylaminobenzaldehyde as a chromogen substrate as described previously (Yago et al., 2011; Selvaggini et al., 2017). Fungal melanin was extracted with 2% NaOH as in Yago et al. (2011). The production of protoplasts was examined after being treated with cell wall-degrading enzymes (CWDEs) as in Chung and Lee (2014).

ACT was purified from culture filtrates of wild-type grown in PDB for 7 days using Amberlite XAD-2 resins (Sigma-Aldrich) and ethyl acetate as described (Kohmoto et al., 1993). The quantity of ACT was calculated and normalized to the dry weight of mycelium as described previously (Wu et al., 2020a).

### Assays for Fungal Virulence

Assays for fungal virulence and ACT toxicity were carried out on detached calamondin (*Citrus mitis* Blanco) leaves. Ten microliter of conidia suspensions (10^5^ conidia/ml) was dropped on the surface of calamondin leaves that were wounded and treated with 0.05% dimethyl sulfoxide (DMSO)/1% ethanol mixture, methanol, 0.2 mM FeCl_3_/0.1% oleic acid (dissolved in ethanol)/5 μM biotin (dissolved in 10% DMSO) mixture, or toxin (10X or 1,000X dilution in methanol) 30 min before inoculation. The treated leaves were kept in a plastic box for 3–5 days for lesion development. Each treatment contained at least four leaves, and experiments were repeated twice showing similar results.

### Microscopy

Fluorescence was examined by a ZOE Fluorescent Cell Imager (Bio-Rad, Hercules, CA, United States) microscope equipped with specific wavelength filters. Red fluorescence from mCherry was visualized using 543 nm excitation and 560–615 nm emission. Red fluorescence after staining with 1 μg/ml MM 4–64 (Enzo, New York, NY, United States) for 30 min was observed using 588 nm excitation and 734 nm emission to track endocytosis. Green fluorescence in hyphae after staining with 40 μM 2'-7'-dichlorofluorescin diacetate (DCFHDA, Sigma-Aldrich) was examined using 504 nm excitation and 529 nm emission. Blue fluorescence in hyphae after staining with 100 μM monodansylcadaverine (MDC, Sigma-Aldrich) was examined using 335 nm excitation and 518 nm emission to identify autophagosomes, amphisomes, and autolysosomes. Fluorescence after staining with 20μM CellTracker^™^ Blue CMAC Dye (Molecular Probes C2110, Thermo Fisher Scientific, Waltham, MA, United States) was visualized using 353 nm excitation and 466 nm emission to detect the vacuoles. Transmission electron microscopy (TEM) using a JEOL JEM-1400 series 120 kV Transmission Electron Microscope (JEOL, Tokyo, Japan) was conducted to examine hyphae/conidia, which were soaked in 2.5% glutaraldehyde and 1% osmium tetraoxide, embedded in LR White Resin, sliced, and stained with uranyl acetate and lead citrate.

### Statistical Analysis

Unless otherwise indicated, all experiments were conducted three times with at least three replicates. The significance of treatments was determined by one-way ANOVA (analysis of variance) and treatment separated by post-hoc Tukey’s HSD (honestly significant difference) test (*p* < 0.05).

## Results

### Localization of Peroxisomes

**Figure 1:**
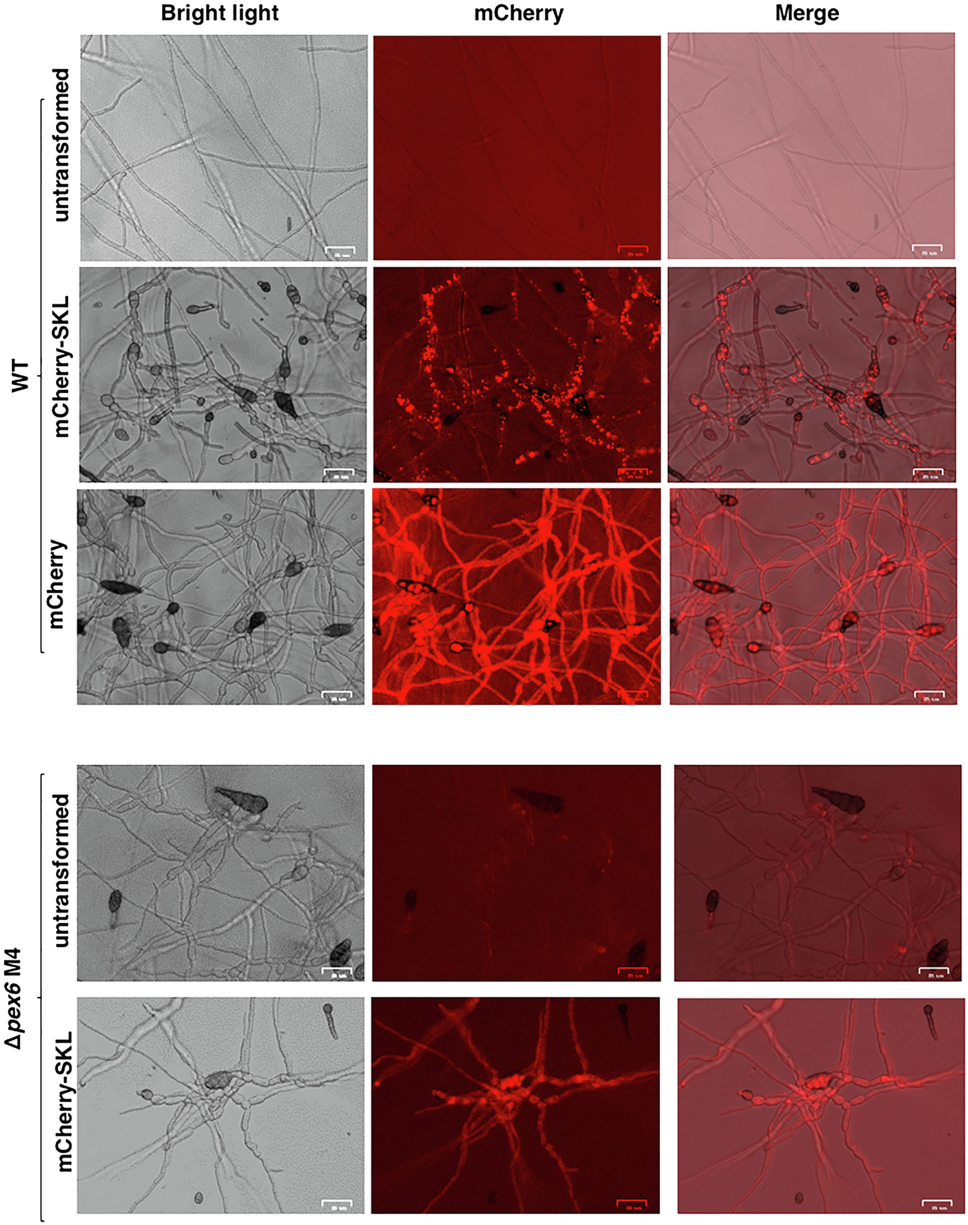
Localization of peroxisomes in *A. alternata*. Fungal strains expressing a mCherry fluorescent protein tagging with or without a conserved tripeptides serine-lysing-leucine (SKL) at the carboxyl terminus. The wild-type strain (WT) expressing mCherry-SKL displays distinct red fluorescent spots. WT expressing mCherry without SKL displays uniform red fluorescence. Fungal strain (Δ*pex6*) impaired for *Pex6* expressing mCherry-SKL displays less distinct fluorescent spots compared to WT expressing the same construct. Bar = 25 μm.

Fluorescence microscopy revealed that the wild-type strain expressing the mCherry-SKL construct displayed distinct red fluorescent spots, indicative of the location of peroxisomes in hyphae (Figure 1). Red fluorescence was barely observed in mature conidia. The wild-type hyphae expressing mCherry without SKL tripeptides resulted in bright, uniform red fluorescence in the hyphal cytoplasm, a stark contrast to the image of hyphae expressing mCherry-SKL. Fluorescence appeared as less distinct patches in the Δ*pex6* hyphae expressing mCherry-SKL compared with fluorescence patterns seen in the wild-type hyphae. No auto red fluorescence was observed in fungal hyphae without expressing mCherry. When conidia prepared from the wild-type/mCherry-SKL were incubated in PDB (a nutrient-rich medium), the intensity of red fluorescence increased with time and was much stronger than those from MM (a nutrient-limiting medium) after a 7-h incubation (data not shown).

### Pex6 Is Required for the Formation of Woronin Bodies, but Not Peroxisome Biogenesis

**Figure 2:**
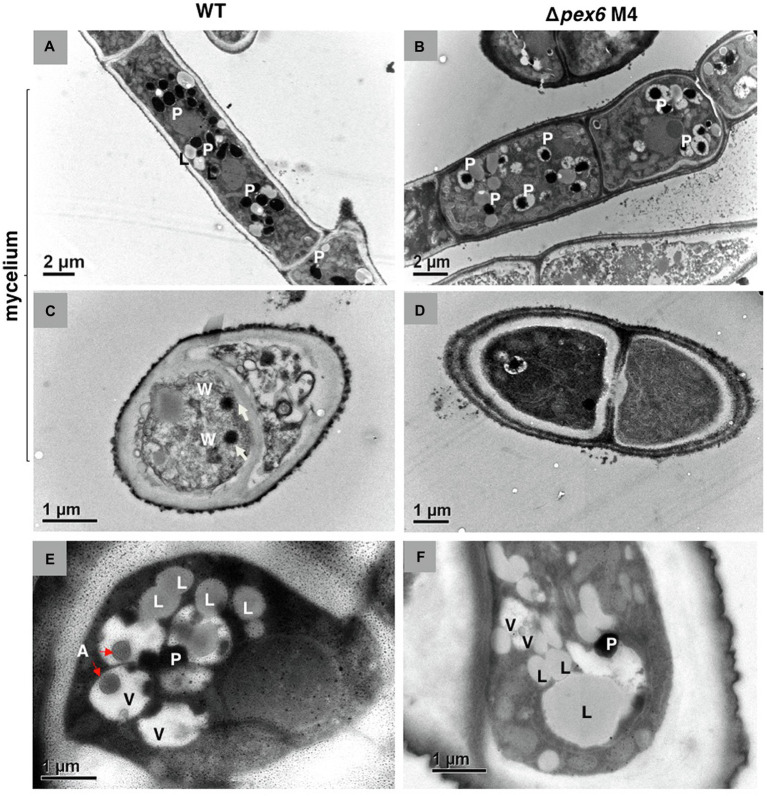
Transmission electron microscopy of *A. alternata* hyphae **(A–D)** and conidia **(E,F)**. Longitudinal **(A)** and cross **(C)** sections of wild-type (WT) hyphae. Longitudinal **(B)** and cross **(D)** sections of Δ*pex6* hyphae. P, peroxisome; L, lipid body; W, Woronin body; A, autophagic body; and V, vacuole.

Transmission electron microscopy revealed that peroxisomes appeared as dark spots in the cytosol (Figures 2A,B). Woronin bodies were occasionally observed near septa in hyphae of wild-type (Figure 2C) and never observed in Δ*pex6* hyphae (Figure 2D). Similarly, autophagic bodies appearing as a gray spot within the vacuole were observed primarily in conidia of wild-type (Figure 2E) and rarely observed in Δ*pex6* conidia (Figure 2F).

### Pex6 Deficiency Reduces Cell Wall Integrity Regulated by the Slt2 MAP Kinase

**Figure 3:**
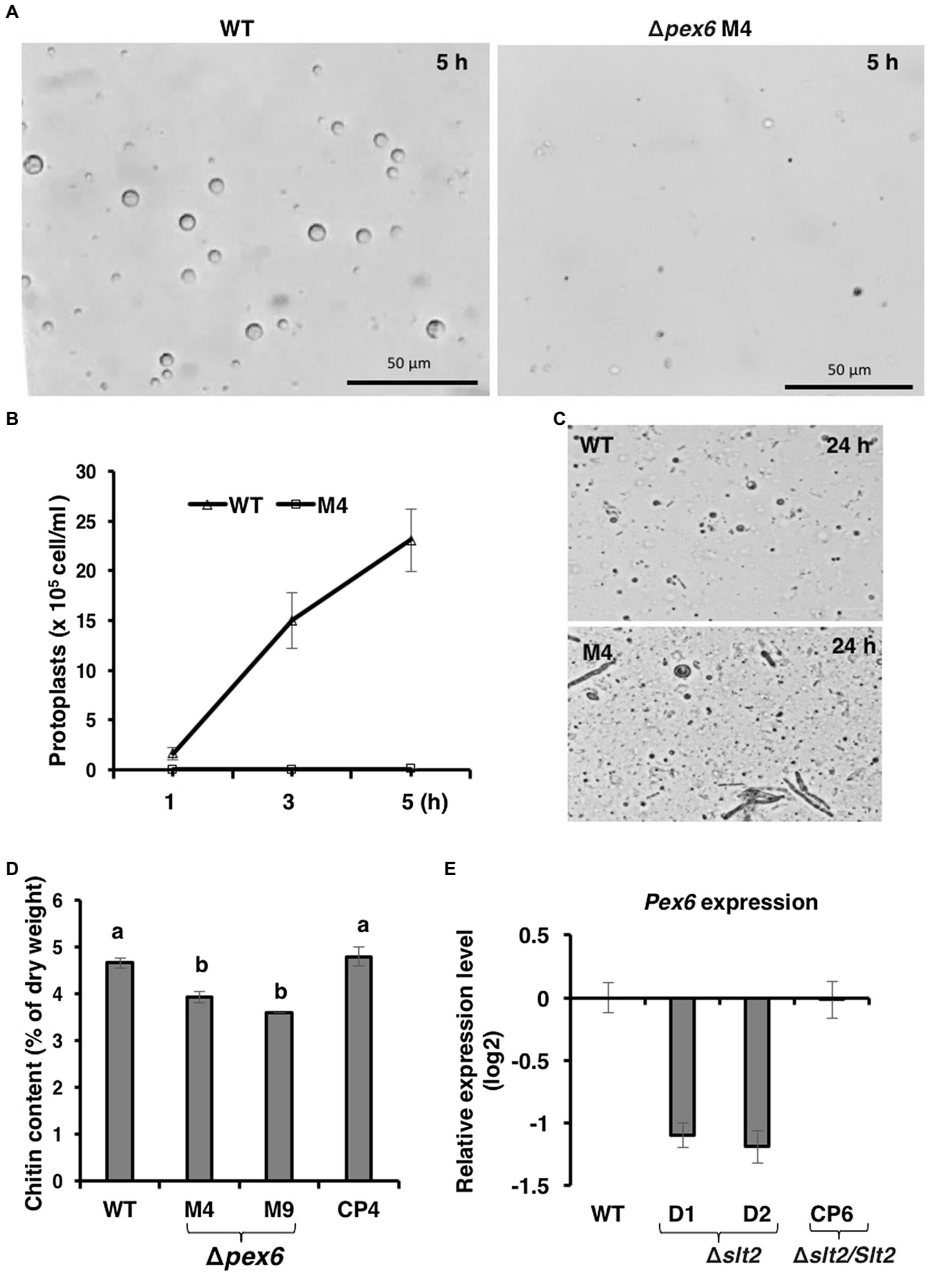
*Pex6* encoding an ATPase-associated (AAA) protein is required for cell wall integrity of *A. alternata*. **(A)** Release of fungal protoplasts from the wild-type (WT) and the Δ*pex6* M4 strains after being treated with cell wall-degrading enzymes (CWDEs) for 5 h. **(B)** Quantitative analysis of protoplast release from WT and M4 over time. **(C)** A prolonged incubation (24 h) of fungal strains with CWDEs. **(D)** Comparison of chitin among WT, two Δ*pex6* strains (M4 and M9) and the CP4 complementation strain re-expressing a functional copy of *Pex6* in M4. Significance of difference between means was analyzed using Tukey’s HSD post-hoc test (*p* ≤ 0.05). Means (*n* = 100) followed by the same letters are not significantly different. **(E)** Quantitative real-time (qRT)-PCR analysis of the *Pex6* transcripts in WT, two Δ*slt2* mutants (D1 and D2), and the CP6 complementation strain re-expressing a functional copy of *Slt2* in D1.

The integrity of fungal cell wall was examined by the production of protoplasts after being treated with CWDEs. Wild-type hyphae released abundant protoplasts 3–5 h after incubation with CWDEs (Figures 3A,B). Very few protoplasts were observed from Δ*pex6* hyphae. A prolonged incubation of Δ*pex6* hyphae with CWDEs resulted in the accumulation of cell debris (Figure 3C). Δ*pex6* had lower chitin contents than the wild-type and the CP4 complementation strains (Figure 3D). There was no significant difference in melanin in the cell wall of wild-type, Δ*pex6*, and CP4. Quantitative RT-PCR analysis revealed that expression of *Pex6* was downregulated in fungal strains (Δ*slt2*) carrying a mutation in the Slt2 MAP kinase (Figure 3E), which has been implicated in the maintenance of cell wall integrity in *A. alternata* (Yago et al., 2011). The transcript level of *Pex6* in the fungal strain (Δ*slt2*/*Slt2*) expressing a functional copy of the Slt2-coding gene was similar to that of wild-type.

### Pex6 Contributes to Siderophore Biosynthesis

**Figure 4:**
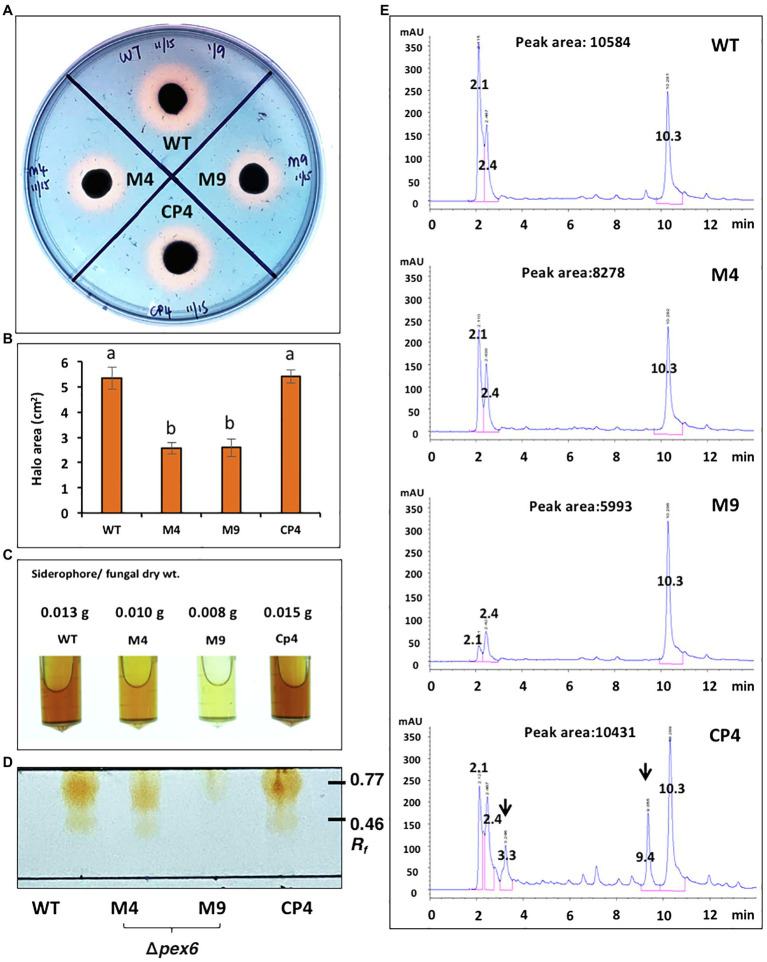
Pex6 is involved in the production of siderophores in *A. alternata*. **(A)** Chrome azurol S (CAS) assays for the production of siderophores by wild-type (WT), Δ*pex6* (M4 and M9), and the CP4 complementation strains. **(B)** Quantitative measurement of the area of orange-yellow halos (minus fungal colony). **(C)** Siderophores purified by Amberlite XAD-16 resins from culture filtrates of fungal strains. **(D)** TLC analysis of siderophores. **(E)** HPLC analysis of siderophores purified from fungal strains. The identities of two additional peaks (indicated by arrows) found in the sample prepared from CP4 remain unknown. Peak areas are the sum of three major peaks (retention time: 2.1, 2.4, and 10.3 min).

Ferric chloride (FeCl_3_) partially restored the defective growth of Δ*pex6*, suggesting the involvement of Pex6 in iron uptake. The production of siderophores (iron chelators) was assessed by culturing fungal strains on a medium containing a chrome azurol S dye and HDTMA. Wild-type formed defined orange halos around colonies, indicative of siderophore production (Figure 4A). Δ*pex6* produced orange halos significantly smaller than those produced by wild-type (Figure 4B). The CP4 strain produced orange halos with areas comparable to those produced by wild-type. Samples purified from culture filtrates of wild-type and CP4 reacted with FeCl_3_ and formed a dark red color, which was in stark contrast to samples purified from Δ*pex6* (Figure 4C). TLC analysis of purified siderophores yielded reddish orange bands at *R_f_* 0.46–0.77 (Figure 4D). The results revealed that siderophores purified from culture filtrates of Δ*pex6* resulted in band intensities much fainter than those purified from culture filtrates of wild-type and CP4. LC–MS/MS analyses revealed that the wild-type and the CP4 strains produced coprogen and hydroxycoprogen (Supplementary Figure S1). HPLC analyses identified three major peaks (retention time: 2.1, 2.4, and 10.3 min) from culture extracts prepared fungal strains (Figure 4E). Two additional peaks (retention time: 3.3 and 9.4) with unknown identities were identified from culture extracts of CP4. Based on three major peak areas, Δ*pex6*-M4 and M9 produced less siderophores than wild-type and CP4.

**Figure 5:**
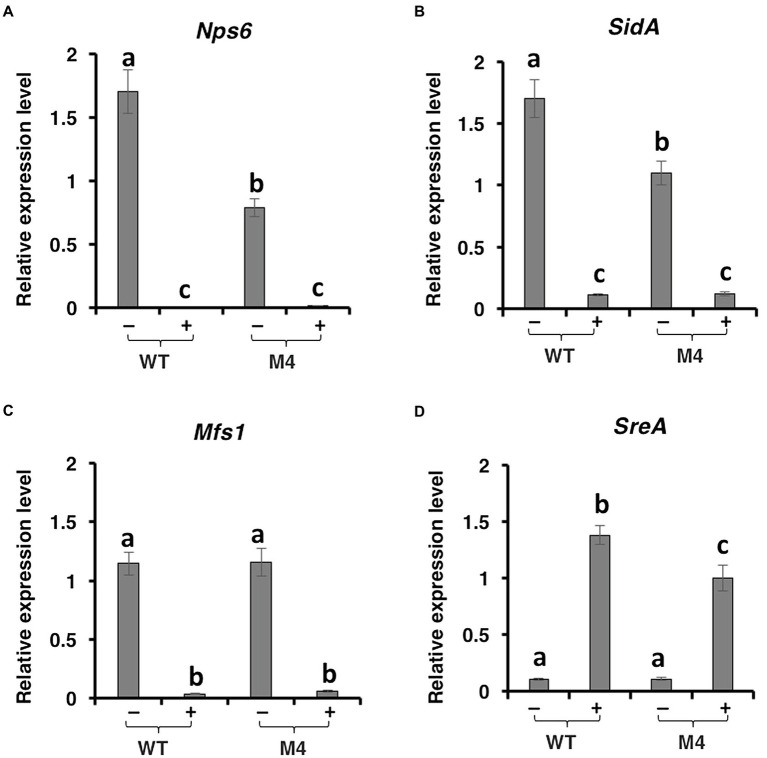
Pex6 impacts the expression of genes involved in the biosynthesis, regulation, and transportation of siderophores in *A. alternata*. Quantitative real-time PCR analysis of the expression of: **(A)** a *Nps6* gene encoding a nonribosomal peptide synthetase, **(B)** a *SidA* gene encoding an L-ornithine N5-oxygenase, **(C)** a *Msf1* gene encoding a major facilitator superfamily (MSF), and **(D)** a *SreA* gene encoding a siderophore repressor in the wild-type (WT) and the ∆*pex6* M4 strains. The relative expression level from three independent reactions was calculated by a comparative CT method (ΔΔCT) in relation to the expression of fungal *β*-tubulin-coding gene.

Quantitative RT-PCR analyses revealed that expression of the *nps6* gene (accession no. JQ973666) encoding a nonribosomal peptide synthetase implicated in the biosynthesis of siderophores was significantly downregulated in Δ*pex6* under iron-depleted conditions (Figure 5A). The *Nps6* gene was not expressed in fungal strains grown on iron-rich medium. Expression of the *SidA* gene (accession no. OWY57903.1) encoding a L-ornithine N5-oxygenase involved in siderophore biosynthesis was also downregulated in Δ*pex6* under iron-depleted conditions (Figure 5B). Under iron-rich conditions, both *Nps6* and *SidA* were barely expressed in wild-type and Δ*pex6*. Expression of the *Mfs1* gene (accession no. OWY46295.1) encoding a MFS transporter was downregulated in both wild-type and Δ*pex6* under iron-rich conditions, and its expression was apparently not affected by the deletion of *Pex6* (Figure 5C). The *SreA* gene (accession no. OWY49902.1) encoding an iron repressor containing two zinc finger DNA-binding domains was highly expressed under iron-rich conditions, and its expression was downregulated in Δ*pex6* (Figure 5D). The *SreA* gene transcript was barely detectable under iron-depleted conditions. The involvement of peroxisomes in the biosynthesis of siderophores was confirmed further by identifying a *p*eroxisomal *t*argeting *s*equence 1 (PTS1) with conserved tripeptide sequence (S/C/A)-(K/R/H)-L at the C terminus of two enzymes: mevalonate-CoA hydrolase (SidH, accession no. OWY42147.1) and transacylase (SidF, accession no. XP_018386017) implicated in the biosynthesis of siderophores.

### Addition of Biotin, Oleic Acid, and Iron Partially Restores Growth Deficiency of Δ*pex6*

**Figure 6:**
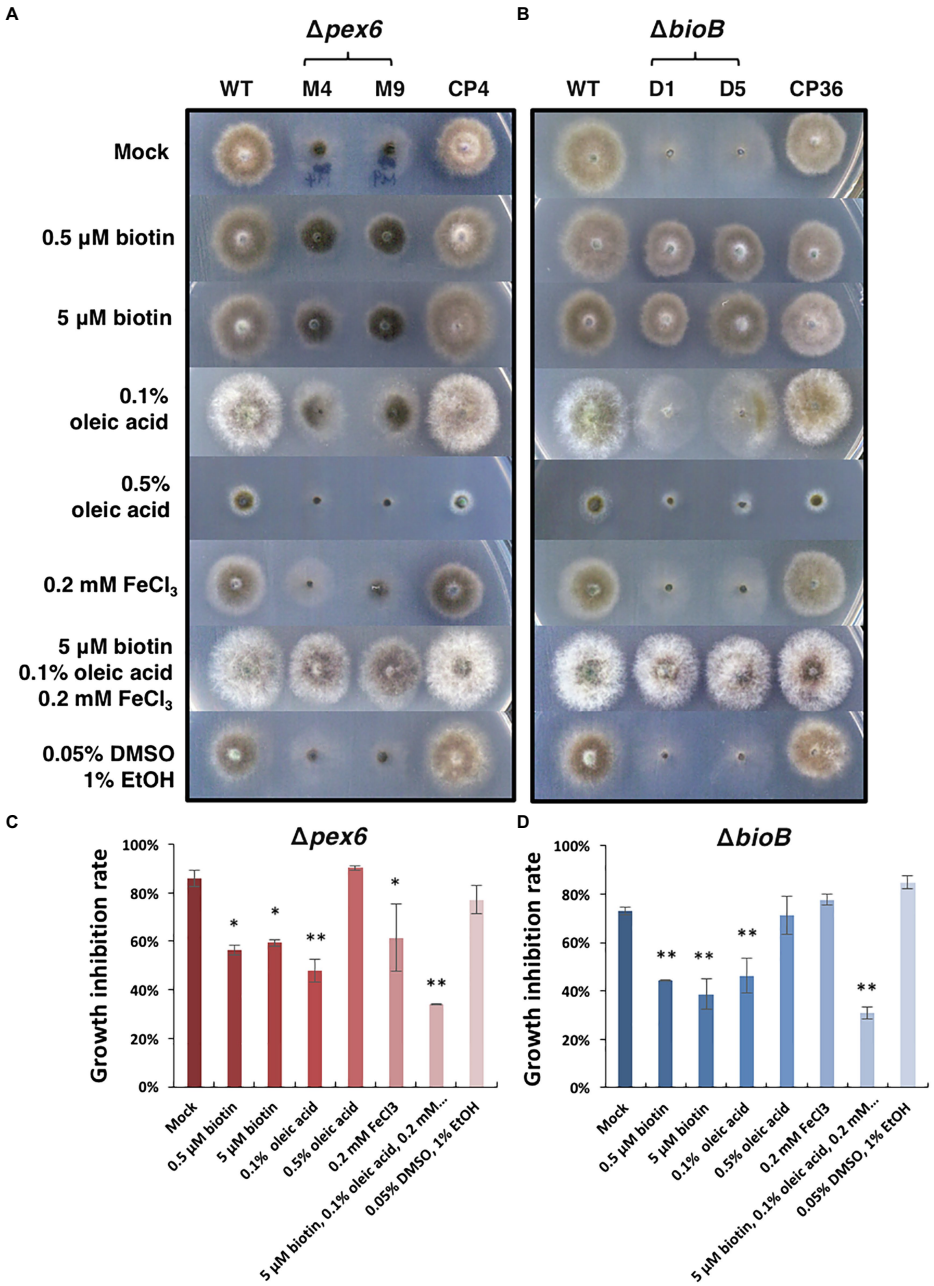
Effects of biotin, oleic acid, and iron on the growth of *A. alternata* strains. **(A)** Growth of the wild-type (WT), the *pex6*-deficient (∆*pex6*-M4 and M9), and the CP4 complementation strains on minimal medium (mock) amended with biotin, oleic acid, and/or FeCl_3_ for 3 days. A combination of biotin, oleic acid, and iron partially restores growth deficiency of Δ*pex6*. **(B)** Growth of the wild-type and the *bioB*-deficient strains (∆*bioB*-D1 and D5) mutated at the biotin synthase (BioB)-coding gene, and the CP36 complementation strain on minimal medium amended with or without biotin, oleic acid, and/or iron. Biotin was dissolved in 10% DMSO, and oleic acid dissolved in 95% ethanol (EtOH). Quantitative analysis of growth reduction of Δ*pex6* **(C)** and Δ*bioB* **(D)** in relation to WT. Means indicated by asterisks are significantly different from the mock control, ^**^p < 0.01, and ^*^p < 0.05.

Δ*pex6* grew appressed and slowly on MM containing glucose as the sole carbon source. Adding biotin (dissolved in DMSO) to MM partially restored Δ*pex6* growth (Figure 6A). Adding 0.1% but not 0.5% oleic acid (dissolved in ethanol) or FeCl_3_ to MM also slightly enhanced the growth of Δ*pex6*. Co-addition of FeCl_3_, oleic acid, and biotin, however, increased Δ*pex6* growth considerably. The *A. alternata* Δ*bioB* strain mutated at the biotin synthase (BioB)-coding gene also grew slowly and appressed on MM. Addition of biotin in MM restored Δ*bioB* growth deficiency (Figure 6B). Addition of 0.1% oleic acid slightly enhanced the growth of Δ*bioB*. As with Δ*pex6*, co-addition of FeCl_3_, oleic acid, and biotin together almost fully restored growth deficiency in Δ*bioB*. Quantitative analysis further confirmed the significance of the treatments (Figures 6C,D).

### A Combination of Biotin, Oleic Acid, and Iron Fails to Restore Δ*pex6* Virulence

**Figure 7:**
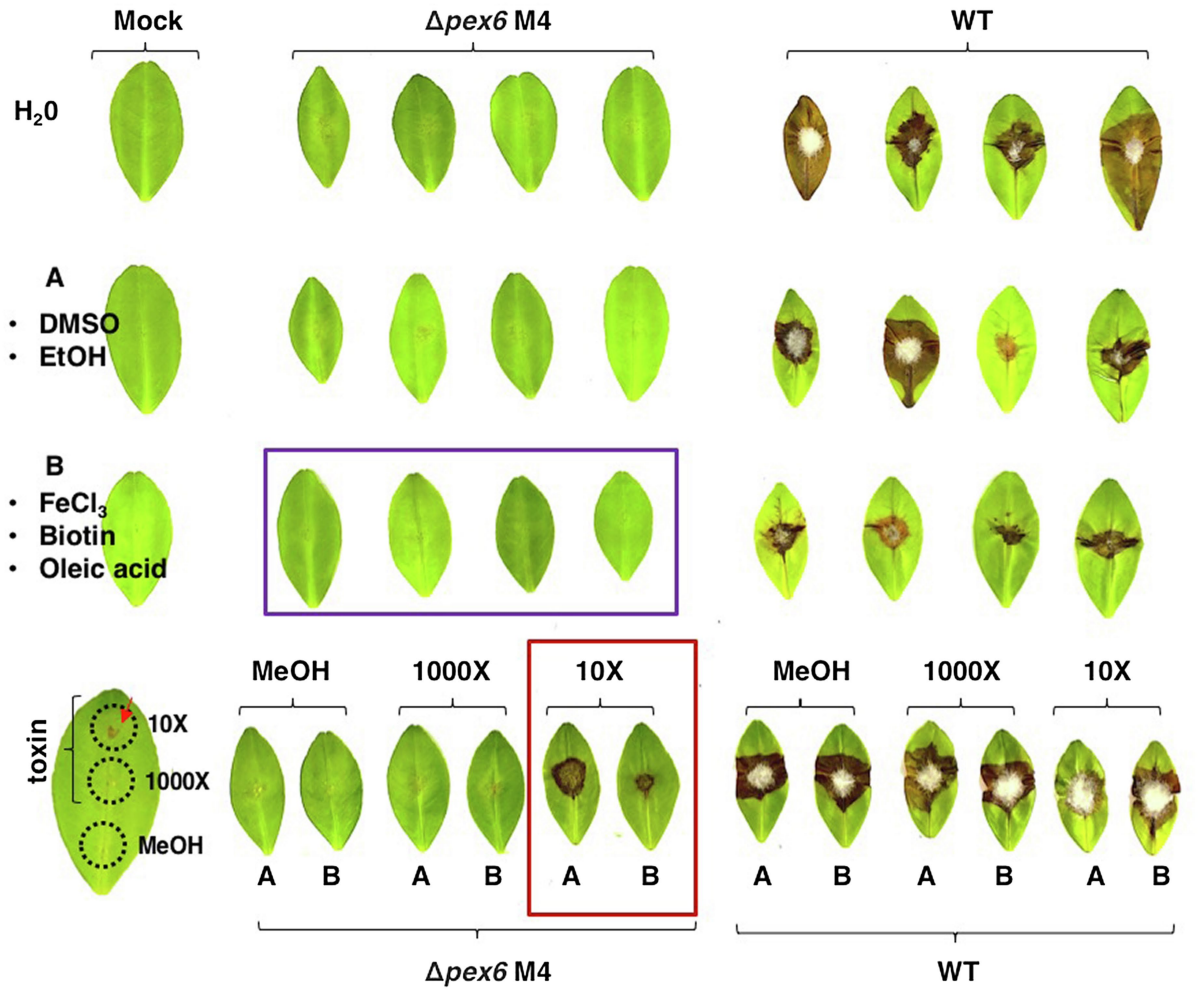
A combination of biotin, oleic acid, and iron fails to restore Δ*pex6* virulence on calamondin leaves. Leaves were wounded and treated with dimethyl sulfoxide (10%, DMSO)/ethanol (EtOH) mixture (an A letter with circle), methanol (MeOH), 0.2 mM FeCl_3_/0.1% oleic acid/0.5 μM biotin mixture (a B letter with circle), or toxin (10X or 1,000X dilution in methanol). After 30 min, 10 μl of conidia suspensions (10^5^ conidia/ml) prepared from wild-type (WT) and ∆*pex6*-M4 were placed on each spot. Leaves treated with water, DMSO/EtOH, MeOH, or toxin alone were used as mock controls.

Since a combination of FeCl_3_, oleic acid, and biotin could restore the growth of Δ*pex6*, experiments were conducted to test if addition of these three compounds together would restore Δ*pex6* virulence on citrus leaves. The results revealed that Δ*pex6* conidia suspensions amended with FeCl_3_, oleic acid, and biotin led to no necrotic lesions on detached calamondin leaves (Figure 7). Addition of the purified ACT (~12 μg crude extracts/g mycelium at a 10X dilution) alone or mixing with FeCl_3_, oleic acid, and biotin in the conidial suspensions prepared from Δ*pex6* partially restored its ability to induce necrotic lesions. ACT at a 1,000X dilution failed to restore Δ*pex6* virulence. Application of the purified ACT alone at a 10X but not 1,000X dilution resulted in necrotic lesions on calamondin leaves that were wounded before application.

### Pex6 Plays a Negative Role in Chemical Resistance

**Figure 8:**
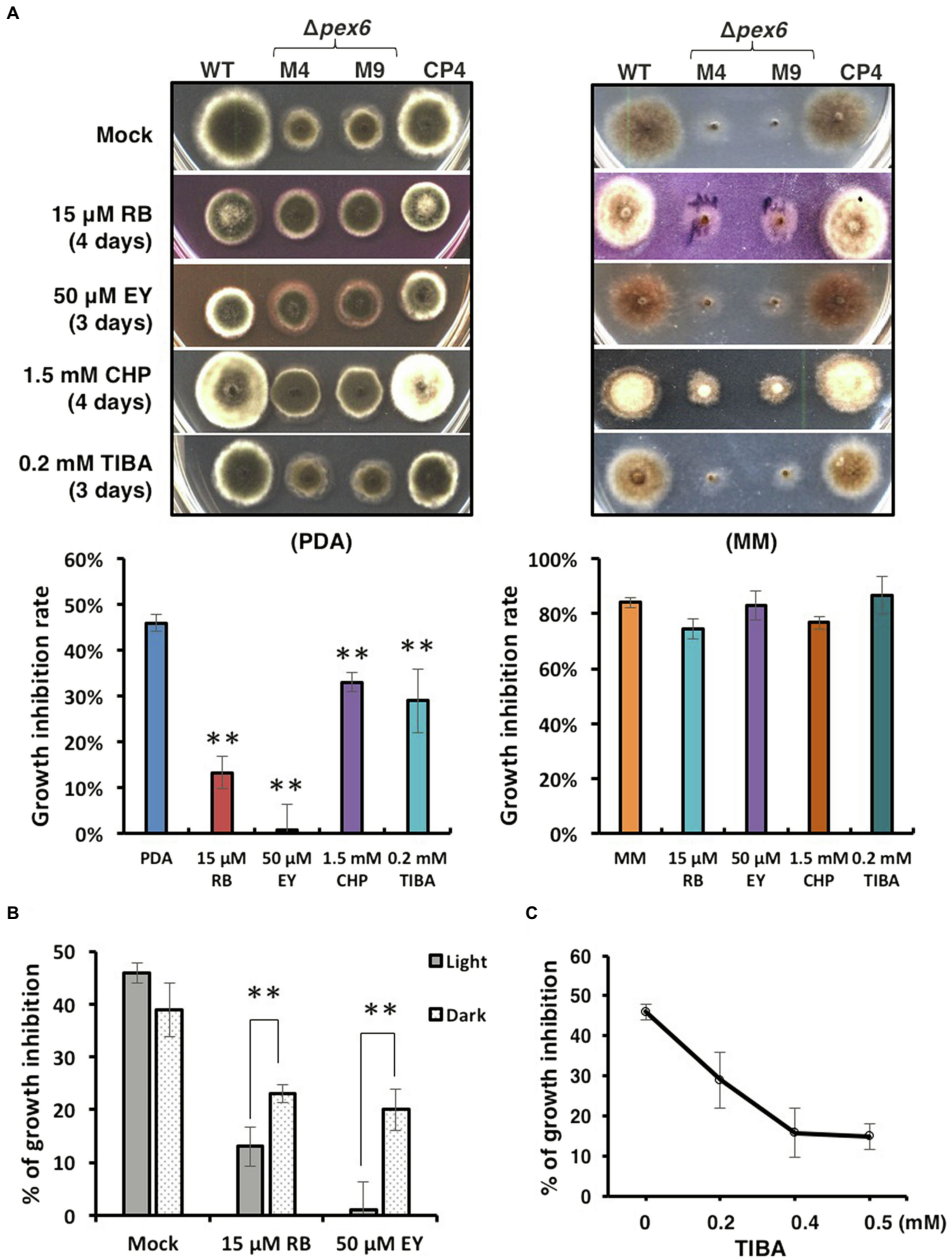
Sensitivity tests. **(A)** Images of the wild-type (WT), the *pex6*-deficient (∆*pex6*-M4 and M9), and the CP4 complementation strains grown on potato dextrose agar (PDA) or minimal medium (MM) amended with rose Bengal (RB), eosin Y (EY), 2-chloro-5-hydroxypyridine (CHP), or 2,3,5-triiodobenzoic acid (TIBA) for 3–4 days. PDA or MM was used a mock control. **(B)** Quantitative analysis of radial growth of WT and ∆*pex6*-M4 on PDA alone (mock) or PDA amended with RB or EY under constant light or in complete darkness for 3–4 days. **(C)** Decreased growth inhibition of ∆*pex6*-M4 in relation to WT on PDA amended with increased concentrations of TIBA. Growth inhibition rates were calculated by dividing the comparative difference of the growth by the wild-type growth and multiplying by 100. Means indicated by asterisks (^**^) are significantly different from one another, *p* < 0.01.

Because Δ*pex6* grew more slowly than wild-type, a decreased growth inhibition rate (below 40%) in Δ*pex6* would indicate an increased resistance to the test compound. Sensitivity tests assayed on PDA revealed that, compared with the wild-type and the CP4 complementation strains, Δ*pex6* increased resistance to rose Bengal (RB, 15 μM), eosin Y (EY, 50 μM), 2-chloro-5-hydroxypyridine (CHP, 1.5 mM), and 2,3,5-triiodobenzoic acid (TIBA, 0.2 mM; Figure 8A). Sensitivity assayed on MM was less conclusive as Δ*pex6* grew poorly on MM. Increased resistance to RB and EY seen in Δ*pex6* grown on PDA was apparently more drastic in light than in darkness (Figure 8B). When grown on PDA amended with different concentrations of TIBA, Δ*pex6* decreased the growth inhibition rate as the concentration of TIBA increased (Figure 8C).

### Degradation of Peroxisomes Is Triggered by Hydrogen Peroxide

**Figure 9:**
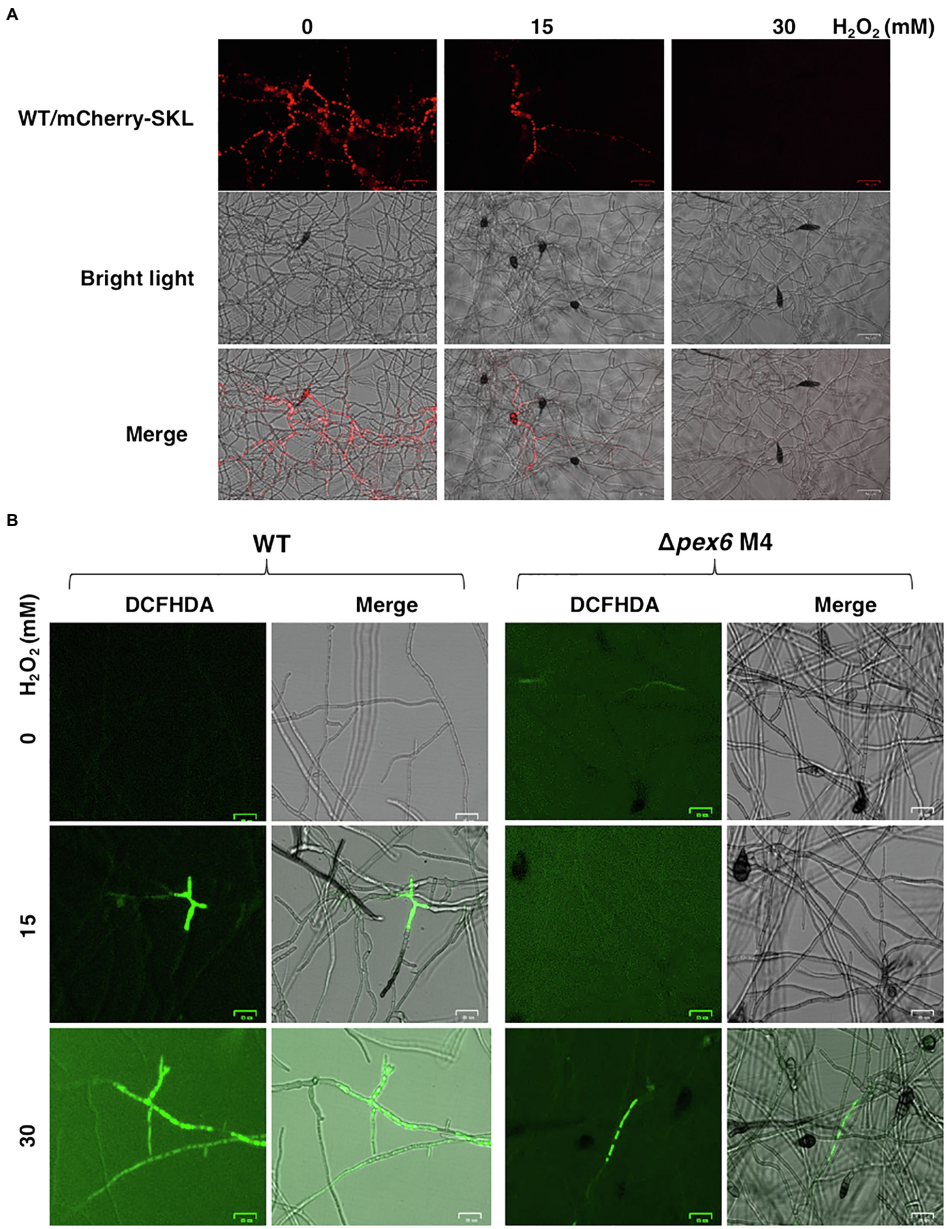
Hydrogen peroxide (H_2_O_2_) impacts the dynamics of peroxisomes, and Pex6 is required for H_2_O_2_ import in *A. alternata*. **(A)** Hyphae of wild-type (WT) expressing a mCherry fluorescent protein tagging with a conserved tripeptides SKL at the carboxyl terminus in response to different concentrations of H_2_O_2_. WT was cultured in potato dextrose broth (PDB) with or without H_2_O_2_ for 4 h and examined by a fluorescent microscope. **(B)** Hyphae of WT and ∆*pex6*-M4 grown in PDB with or without H_2_O_2_ for 2 h were stained with 2'-7'-dichlorofluorescin diacetate (DCFHDA), displaying green fluorescence, indicative cellular accumulation of H_2_O_2_.

**Figure 10:**
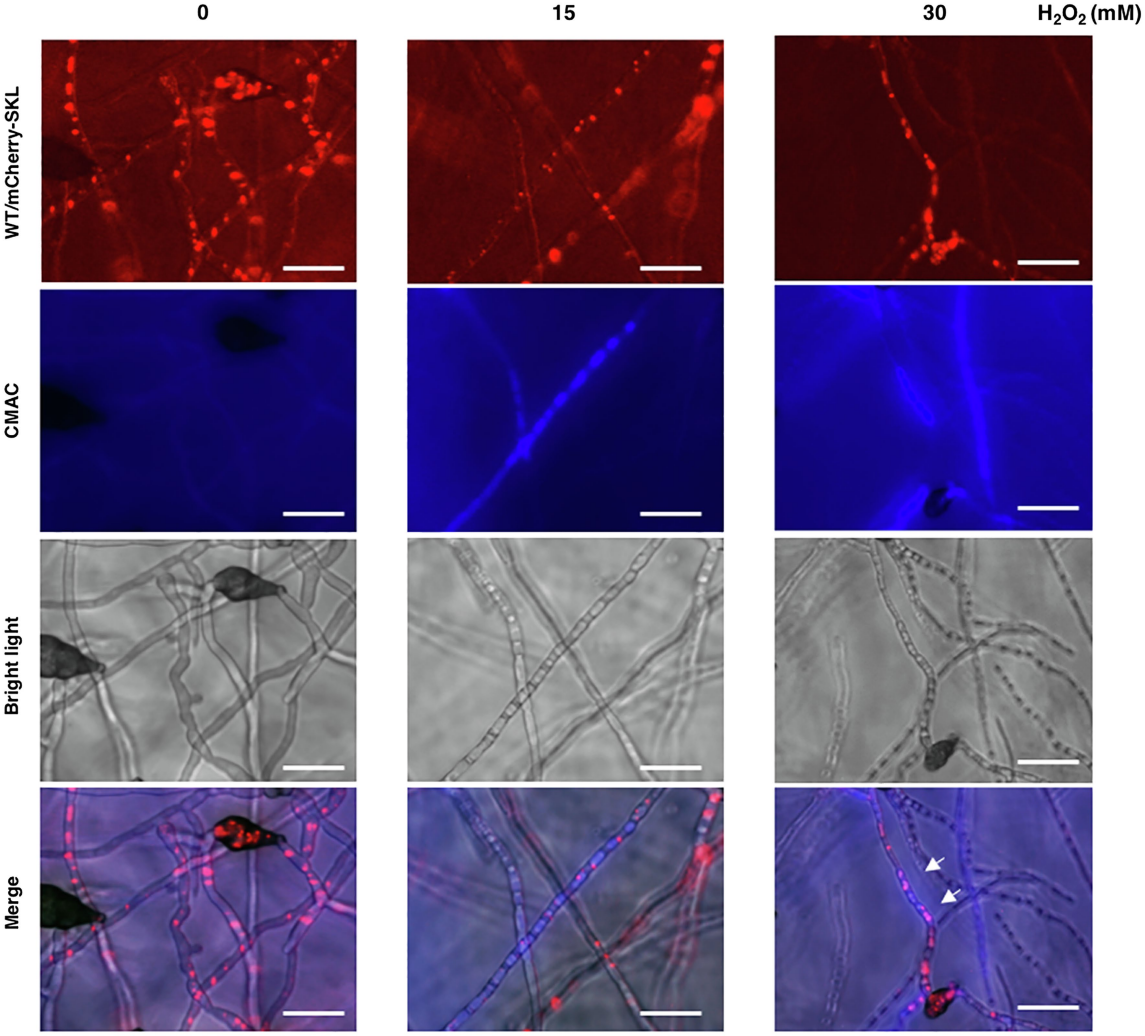
The peroxisomal reporter protein mCherry-SKL is degraded in vacuoles. The wild-type strain expressing mCherry-SKL was treated with different concentrations of H_2_O_2_ in PDB for 4 h, stained with CMAC, and observed by a fluorescent microscope. Co-localization of the mCherry-SKL proteins with vacuoles (indicated by arrows) was observed. The decrease of mCherry-SKL fluorescence was closely correlated with the increase of blue fluorescence emitted from CMAC, implicating the degradation of peroxisomes in vacuoles. Bar = 25 μm.

Previous studies have revealed that the wild-type strain of *A. alternata* could resist to high concentrations of hydrogen peroxide (H_2_O_2_). Fluorescence microscopy revealed that wild-type expressing mCherry-SKL displayed different patterns of fluorescence when grown in PDB with or without H_2_O_2_ (Figure 9A). The intensity and spot of red fluorescence, indicative of the location of peroxisomes, decreased considerably in wild-type hyphae grown in PDB amended with H_2_O_2_. Wild-type hyphae displayed green fluorescence after being exposed to H_2_O_2_ and stained with DCFHDA (Figure 9B). Green fluorescence was observed in Δ*pex6* hyphae after being exposed to a higher concentration (30 mM) of H_2_O_2_ and stained with DCFHDA. Compared with wild-type hyphae, fewer hyphae of Δ*pex6* displayed green fluorescence in response to H_2_O_2_. The decrease of peroxisomes as indicated by mCherry-SKL in fungal cells after H_2_O_2_ treatment occurred in parallel with the formation of vacuoles during autophagy, as evidenced by CMAC staining (Figure 10). The results indicated the localization and degradation of peroxisomes within vacuoles after H_2_O_2_ treatment.

### A Link Between Pex6 and Autophagy

**Figure 11:**
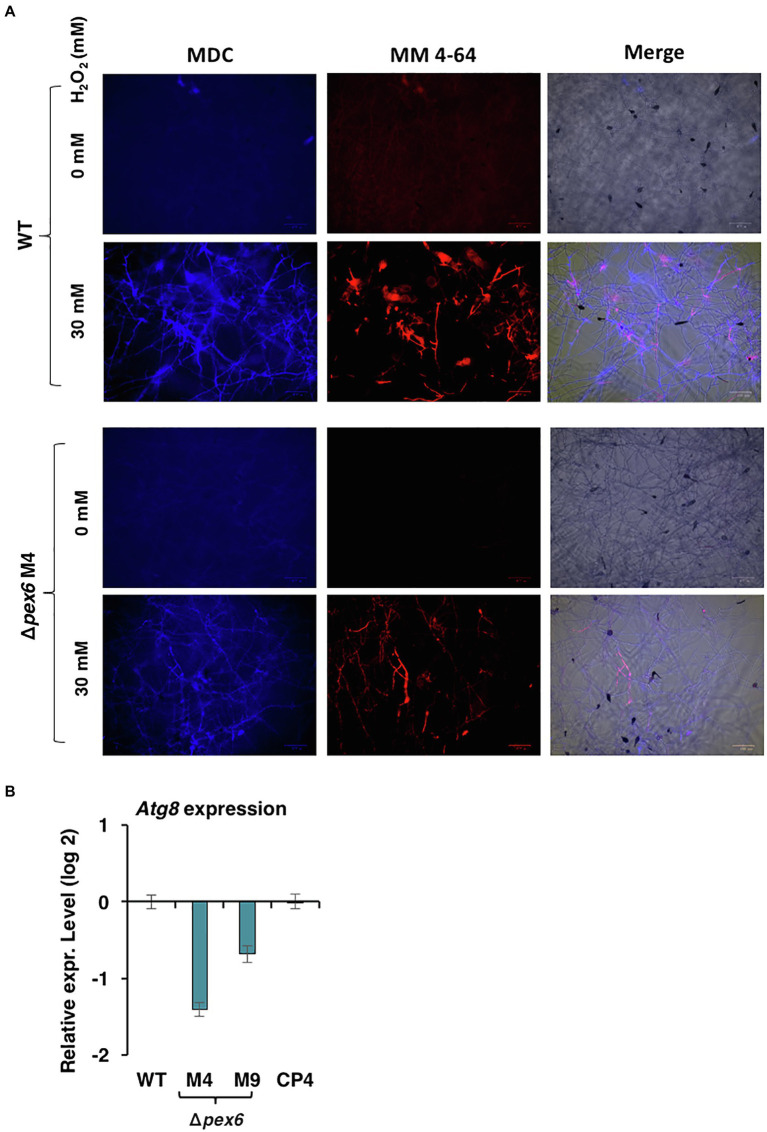
Pex6 impacts autophagy process triggered by hydrogen peroxide (H_2_O_2_) in *A. alternata*. **(A)** Hyphae of WT and ∆*pex6*-M4 grown in PDB with or without H_2_O_2_ for 4 h were co-stained with monodansylcadaverine (MDC) and MM 4–64. Δ*pex6* hyphae display much weaker fluorescence than wild-type, suggesting that Pex6 plays a role in autophagy process. **(B)** Quantitative real-time PCR analysis of the expression of an *atg8* gene encoding an autophagy-related protein in the WT, the *pex6*-deficient (∆*pex6*-M4 and M9), and the CP4 complementation strains.

Double staining with monodansylcadaverine (MDC) and MM 4–64 was used to monitor autophagy process (Sengupta et al., 2011; Sun et al., 2018). Wild-type hyphae displayed blue fluorescence after being exposed to H_2_O_2_ and stained with MDC, many of which were aligned with red fluorescence after MM 4–64 staining (Figure 11A). Δ*pex6* hyphae treated with H_2_O_2_ displayed much weaker fluorescence than wild-type, implicating a role of Pex6 in autophagosome turnover by lysosomes. Quantitative RT-PCR analysis revealed that expression of the *Atg8* gene encoding an autophagy-related protein was downregulated in two Δ*pex6* strains compared with the wild-type and the CP4 complementation strains (Figure 11B).

## Discussion

The host-selective toxin produced by the tangerine pathotype of *A. alternata* is absolutely required for colonization and lesion formation on susceptible citrus cultivars (Kohmoto et al., 1993). In addition, *A. alternata* has to assuage the toxicity of ROS released from plant cells after being killed by the toxin (Lin et al., 2009). In order to better understand the mechanisms of cellular resistance to ROS, a *Pex6* gene encoding a protein responsible for protein import into peroxisomes was deleted in the tangerine pathotype of *A. alternata*. Peroxisomes have been well known to be involved in degradation and synthesis of fatty acids as well as generation and detoxification of hydrogen peroxide (Antonenkov et al., 2010; Freitag et al., 2014). Present studies have shown that Pex6 plays an important role in a wide array of physiological and pathological functions in the tangerine pathotype of *A. alternata* (Wu et al., 2020a).

Proteins carrying a conserved tripeptide sequence S/C/A-K/R/H-L at the C terminus are readily imported to peroxisomes (Heiland and Erdmann, 2005; Brocard and Hartig, 2006). When a mCherry fluorescent protein containing SKL tripeptides was expressed in the wild-type strain of *A. alternata*, intense punctate fluorescence, indicative of the location of peroxisomes, was observed within hyphae. Examination of the strain expressing mCherry-SKL revealed that peroxisomes were kinetic organelles, and their numbers varied in response to nutrient availability in environment. The numbers of peroxisomes were also influenced by H_2_O_2_ (Figure 9A) and perhaps other environmental stress as well. Hyphae of Δ*pex6* expressing mCherry-SKL displayed less distinct red fluorescence. TEM observations revealed that the numbers of peroxisomes in Δ*pex6* were not significantly different from those of wild-type, indicating that *Pex6* deletion resulted in defective protein transport into peroxisomes rather than peroxisomal biogenesis in *A. alternata*.

Although sensitivity assays revealed that Pex6 plays no roles in cellular resistance to H_2_O_2_, the role of peroxisomes in ROS resistance remains uncertain. Pex6 is involved in protein import into peroxisomes and apparently plays no roles in peroxisomal biogenesis as deletion of *Pex6* had no impact on the numbers of peroxisomes. However, maintaining normal functions of protein import into peroxisomes *via* Pex6 was shown to be critical for the formation of Woronin bodies, cell wall integrity, the biosynthesis of biotin, siderophores, and ACT, as well as the uptake and accumulation of H_2_O_2_. Pex6 also plays an important role in spore germination, cellular viability, the formation of appressorium-like structures and lipid bodies, and virulence in the tangerine pathotype of *A. alternata* (Wu et al., 2020a). The diverse functions of Pex6 illustrate the central role of peroxisomes in fungal growth and development as well as pathogenesis.

Fungal cell wall is a complex structure composed of chitin, glucans, glycoproteins, and pigments. Synthesis and remodeling of cell wall involving Golgi, plasma membrane, and cell wall itself are highly regulated (Gow et al., 2017). The exact role of peroxisomes in the biosynthesis of fungal cell wall remains unknown. The *pex6*-deficient strain increases sensitivity to chitin-binding compounds, Congo red and calcofluor white (Wu et al., 2020a). TEM observations also revealed that the *pex6*-deficient strain has thinner cell walls than wild-type, suggesting a malfunction of cell walls. Deformation of cell wall integrity in Δ*pex6* might be one of the reasons contributing to its lower viability (Wu et al., 2020a). The current study revealed that the *pex6*-deficient strain has lower chitin content of cell walls compared to wild-type. However, upon being exposed to CWDEs for 5 h, the *pex6*-deficient strain hyphae failed to yield abundant protoplasts. This is probably due to the lack of ability to maintain the membrane integrity of protoplasts because a prolonged incubation (24 h) of Δ*pex6* hyphae with CWDEs resulted in numerous cell debris. Nevertheless, maintaining proper functions of peroxisomes *via* Pex6-mediated protein import is critical for structure and biogenesis of the cell walls of *A. alternata*. The involvement of peroxisomes in cell wall integrity has also been reported in *Fusarium graminearum* and *Magnaporthe oryzae* (Goh et al., 2011; Li et al., 2014; Zhang et al., 2019). One of the significant findings of the current study is the establishment of a connection between peroxisomes and the mitogen-activated protein kinase Slt2. The Slt2-mediated signaling pathway plays a central role in maintaining cell wall integrity in fungi (Xu, 2000). The expression of *Pex6* is significantly downregulated in the *slt2*-deficient strains, suggesting that Pex6 is either directly or indirectly regulated by Slt2. This association has never been reported in fungi. Both the *pex6*- and the *slt2*-deficient strains have common phenotypic characteristics. As with Δ*pex6*, deletion of the Slt2-coding gene results in a mutant strain that increases sensitivity to Congo red and calcofluor white, reduces cell wall thickness, and decreases chitin contents (Yago et al., 2011). It is likely that some of the proteins involved in chitin biosynthesis are likely localized in peroxisomes.

*Alternaria alternata* is capable of producing siderophores to acquire iron from environment (Chen et al., 2013). Siderophore-mediated iron uptake is required for growth, and iron plays an important role in cellular resistance to ROS and fungal virulence. Previous studies have revealed that biosynthesis and transport of siderophores are positively influenced by the redox responsive Yap1 transcription regulator (Lin et al., 2009), the high osmolarity-glycerol 1 (Hog1) MAP kinase (Lin and Chung, 2010), the NADPH oxidases (Nox; Yang and Chung, 2012, 2013), the MFS transporters (Chen et al., 2017; Lin et al., 2018), and the nascent polypeptide-associated complex subunit α (Wang et al., 2020), and are negatively regulated by the GATA zinc finger-containing SreA transcription regulator (Chung et al., 2020) in *A. alternata*. Biosynthesis of siderophores is apparently associated with peroxisomes as the *pex6*-deficient mutant accumulates much less siderophores than wild-type. Indeed, Pex6 impacted the expression of genes involved in the biosynthesis and transport of siderophores exclusively under iron-depleted conditions. Moreover, examination of enzymes involved in siderophore biosynthesis for the presence of peroxisomal targeting sequence (PTS) revealed that two enzymes, mevalonate-CoA hydrolase (SidH) and transacylase (SidF), contain conserved SKL tripeptides at each of the C termini. The results indicate that biosynthesis of siderophores, at least in part, occurs in peroxisomes. A link between siderophore biosynthesis and peroxisomes has also been reported in *Aspergillus* spp. (Gründlinger et al., 2013).

Biotin serves as a cofactor of many enzymes involved in the gluconeogenesis, lipid biosynthesis, and amino acid metabolism. As with many fungi and plants (Magliano et al., 2011; Tanabe et al., 2011; Maruyama et al., 2012; Maruyama and Kitamoto, 2013), the production of biotin is associated with peroxisomes in *A. alternata* (Wu et al., 2020b). Both *pex6*- and *bioB* (encoding a biotin synthase)-deficient strains showed biotin auxotrophy and failed to utilize oleic acid efficiently. Oleic acid has been shown to induce the biogenesis of peroxisomes in yeasts (Hutchins et al., 1999; Sibirny, 2016). Exogenous addition of biotin, oleic acid, and FeCl_3_ together could almost fully restore the growth of both *pex6*- and *bioB*-deficient strains, indicating that growth deficiency observed in both mutants is caused by the defective functions of peroxisomes. The results also confirm the important role of peroxisomes in the biosynthesis of biotin and siderophores, as well as lipid metabolism. Although co-addition of biotin, oleic acid, and FeCl_3_ could restore growth deficiency of Δ*pex6*, they failed to restore fungal full virulence. Adding purified toxin, biotin, oleic acid, and FeCl_3_, however, could only partially restore Δ*pex6* virulence. The results clearly indicate that a dynamic function of peroxisomes in the biosynthesis of host-selective toxin is critical for *A. alternata* pathogenesis to citrus. Alternatively, in addition to toxin biosynthesis, peroxisomes may regulate other yet unidentified functions, which are required for fungal pathogenesis.

The *pex6*-deficient mutant displayed wild-type sensitivity to H_2_O_2_, *tert*-butyl-hydroperoxide and potassium superoxide (KO_2_), suggesting that the Pex6-mediated protein import into peroxisomes plays no roles in resistance to peroxide and superoxide stress (Wu et al., 2020a). However, the *pex6*-deficient mutant increased resistance to two singlet oxygen-generating compounds, rose Bengal and eosin Y, particularly under light. Rose Bengal and eosin Y are photosensitizing compounds, which can absorb light energy and react with oxygen to generate extremely toxic singlet oxygen (Amat-Guerri et al., 1990). The targets of singlet oxygen in cells include nucleic acids, proteins, and lipids. It remains unclear why deletion of *Pex6* leads to elevated resistance to singlet oxygen. In addition to rose Bengal and eosin Y, the *pex6*-deficient mutant increased resistance to CHP and TIBA, both of which have been shown to increase hyphal branching, and inhibit growth and conidial formation and germination in *A. alternata* (Chen et al., 2017). CHP could interact with H_2_O_2_ to form superoxide and hydroxyl radicals in the presence of Cu^2+^ (Watanabe et al., 1998). Whether or not TIBA could generate singlet oxygen or other ROS remains uncertain. Peroxisomes have long been thought to be involved in the generation and detoxification of H_2_O_2_ (Schrader and Fahimi, 2006; Antonenkov et al., 2010). Our results have shown that impairment of the peroxisomal protein import pathway in *A. alternata* led to a decreased sensitivity to singlet oxygen-generating compounds but had no effects on cellular resistance to H_2_O_2_. The decreased sensitivity to singlet oxygen-generating compounds as a result of impaired functions of peroxisomes has never been reported in fungi.

When the wild-type strain of *A. alternata* was exposed to H_2_O_2_, an accumulation of H_2_O_2_ accompanying with a reduction of peroxisomes was observed in hyphae. Although the wild-type strain accumulated H_2_O_2_ in hyphae upon exposure to this oxidative stress, deletion of *pex6* prevented import or accumulation of H_2_O_2_ in hyphae. Transport of H_2_O_2_ into cells is mediated by aquaporins and influenced by membrane lipids (Bienert et al., 2006). The exact mechanism of Pex6-associated H_2_O_2_ import or accumulation remains unknown. H_2_O_2_ may trigger the pexophagy process, which facilitates degradation of peroxisomes (Sakai et al., 2006; Manjithaya et al., 2010a; Oku and Sakai, 2010; Germain and Kim, 2020). This assumption was supported by mCherry-SKL and co-staining with CMAC of hyphae after being treated with H_2_O_2_ (Figure 10). The results suggested that H_2_O_2_ may induce the turnover of peroxisomes *via* an autophagy-associated mechanism termed pexophagy in wild-type. Moreover, we have observed that Δ*pex6* hyphae after being treated with H_2_O_2_ and co-stained with MDC and MM 4–64 resulted in weaker fluorescence compared to wild-type hyphae (Figure 11), and it might suggest that Pex6 likely plays a role in autophagosome turnover by lysosomes. The exportomer components consisting of Pex6, Pex1, and Pex15 have been shown to suppress pexophagy in the budding yeast (Nuttall et al., 2014) and mammalian cells (Law et al., 2017). Our results have also shown that the expression of *Pex6* is positively regulated by the Slt2 MAP kinase, implicating a possible involvement of Pex6 in pexophagy. The Slt2 MAP kinase is required for pexophagy in the budding yeast (Manjithaya et al., 2010b). However, the mechanism of how Pex6 impacts autophagy or pexophagy in *A. alternata* remains unclear and warrants further research.

**Figure 12:**
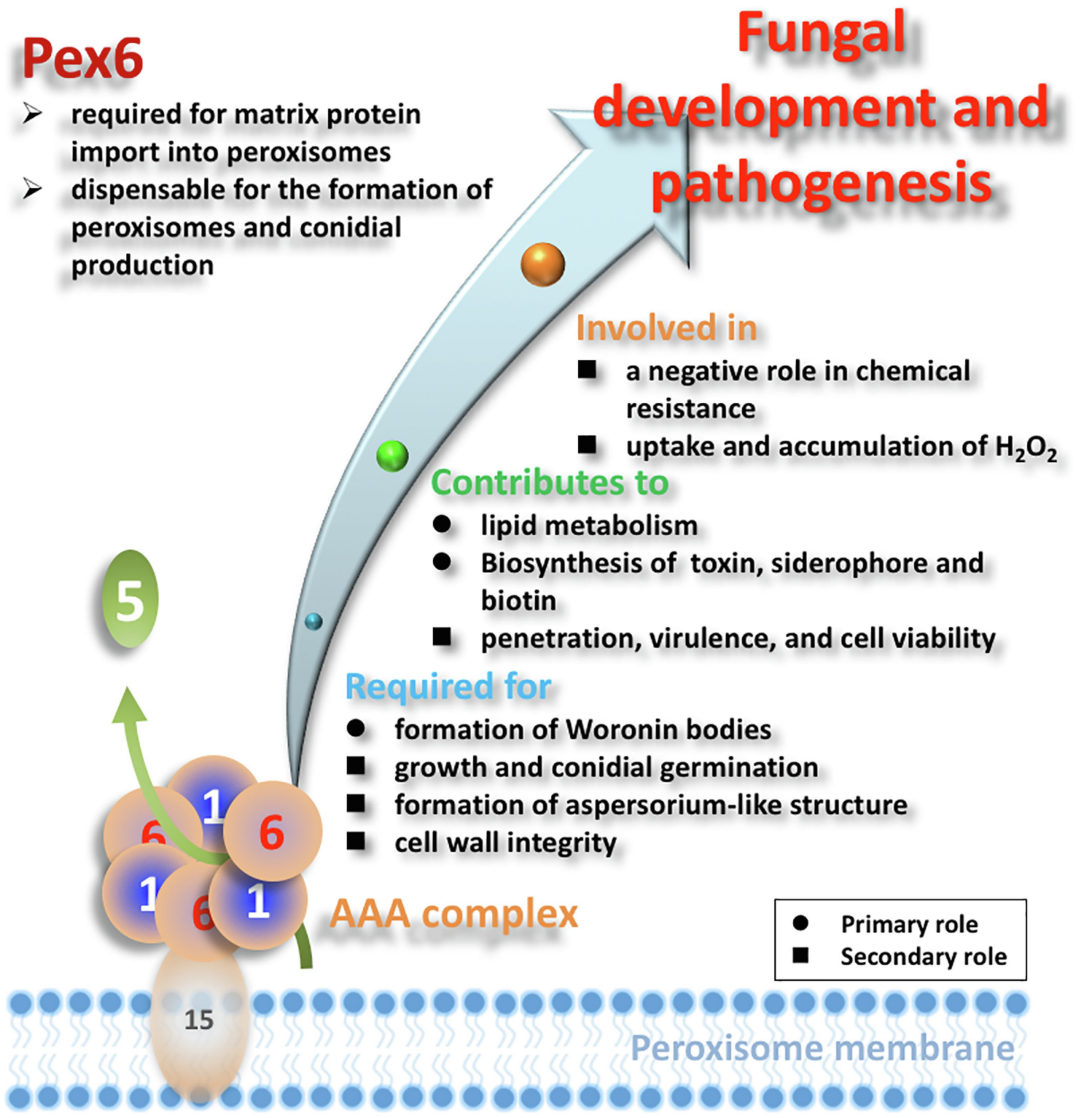
Schematic illustration of the overall functions of Pex6 in *A. alternata*. Matrix proteins are presumably imported by a Pex5 receptor, and the Pex1/Pex6 AAA complex attached to membrane *via* Pex15 is involved in recycling the receptor proteins. Black circle (●) and black square (■) indicate the primary and secondary roles of peroxisomes, respectively.

In conclusion, peroxisome is a dynamic organelle, whose biogenesis and degradation are tightly regulated in response to the environment (Platta and Erdmann, 2007a; Smith and Aitchison, 2009). We have found that the number of peroxisomes was impacted by nutrients and oxidative stress, and associated with the pexophagy process. Peroxisomes have been demonstrated to be directly or indirectly involved in a wide array of metabolic and pathological processes, including cell wall integrity, siderophore-mediated iron uptake, biosynthesis of biotin and host-selective toxin, and virulence in *A. alternata* (Figure 12). Moreover, this study has established a link between Pex6, cell wall integrity, and the Slt2 MAP kinase. Another significant finding of this study is the revelation of a negative role of the Pex6-mediated protein import into peroxisomes in resistance to 2,3,5-triiodobenzoic acid, 2-chloro-5-hydroxypyridine, and singlet oxygen-generating compounds. These findings may have important implications for better understanding the role of peroxisomes in resistance to environmental stress.

## Data Availability Statement

The original contributions presented in the study are included in the article/Supplementary Material, further inquiries can be directed to the corresponding authors.

## Author Contributions

P-CW and K-RC designed and performed the experiments, acquired the data, and wrote the main manuscript text. P-CW, Y-KC, JY, and K-RC analyzed and interpreted the data. All authors contributed to the article and approved the submitted version.

### Conflict of Interest

The authors declare that the research was conducted in the absence of any commercial or financial relationships that could be construed as a potential conflict of interest.

## References

[ref1] Amat-Guerri F. López-González M. M. C. Martínez-Utrilla R. Sastre R. (1990). Singlet oxygen photogeneration by ionized and un-ionized derivatives of rose Bengal and eosin Y in diluted solutions. J. Photochem. Photobiol. A Chem. 53, 199–210.

[ref2] Antonenkov V. D. Grunau S. Ohlmeier S. Hiltunen J. K. (2010). Peroxisomes are oxidative organelles. Antioxid. Redox Signal. 13, 525–537. 10.1016/j.bbamcr.2006.09.006 19958170

[ref3] Bartoszewska M. Opaliński Ł. Veenhuis M. van der Klei I. J. (2011). The significance of peroxisomes in secondary metabolite biosynthesis in filamentous fungi. Biotechnol. Lett. 33, 1921–1931. 10.1007/s10529-011-0664-y 21660569 PMC3173629

[ref4] Bienert G. P. Schjoerring J. K. Jahn T. P. (2006). Membrane transport of hydrogen peroxide. Biochim. Biophys. Acta 1758, 994–1003. 10.1016/j.bbamem.2006.02.015 16566894

[ref5] Brocard C. Hartig A. (2006). Peroxisome targeting signal 1: is it really a simple tripeptide? Biochim. Biophys. Acta. 1763, 1565–1573. 10.1016/j.bbamcr.2006.08.022 17007944

[ref6] Chen L.-H. Lin C.-H. Chung K.-R. (2012). Roles for SKN7 response regulator in stress resistance, conidiation and virulence in the citrus pathogen *Alternaria alternata*. Fungal Genet. Biol. 49, 802–813. 10.1016/j.fgb.2012.07.006 22902811

[ref7] Chen L.-H. Lin C.-H. Chung K.-R. (2013). A nonribosomal peptide synthetase mediates siderophore production and virulence in the citrus fungal pathogen *Alternaria alternata*. Mol. Plant Pathol. 14, 497–505. 10.1111/mpp.12021 23438010 PMC6638914

[ref8] Chen L.-H. Tsai H.-C. Chung K.-R. (2017). A major facilitator superfamily transporter-mediated resistance to oxidative stress and fungicides requires Yap1, Skn7, and MAP kinases in the citrus fungal pathogen *Alternaria alternata*. PLoS One 12:e0169103. 10.1371/journal.pone.0169103 28060864 PMC5218470

[ref9] Chen L.-H. Yang S. L. Chung K.-R. (2014). Resistance to oxidative stress via regulating siderophore-mediated iron-acquisition by the citrus fungal pathogen *Alternaria alternata*. Microbiology 160, 970–979. 10.1099/mic.0.076182-0 24586035

[ref10] Chung K.-R. (2013). Mitogen-activated protein kinase signaling pathways of the tangerine pathotype of *Alternaria alternata*. MAP Kinase 2:e4. 10.4081/mk.2013.e4

[ref11] Chung K.-R. Lee M.-H. (2014). “Split marker-mediated transformation and targeted gene disruption in filamentous fungi.” in Genetic Transformation Systems in Fungi. *Vol*. 2. eds. van den berg M. A. Maruthachalam K. (Switzerland: Springer International Publishing), 175–180.

[ref12] Chung K.-R. Shilts T. Li W. Timmer L. W. (2002). Engineering a genetic transformation system for *Colletotrichum acutatum*, the causal fungus of lime anthracnose and postbloom fruit drop. FEMS Microbiol. Lett. 213, 33–39. 10.1111/j.1574-6968.2002.tb11282.x 12127485

[ref13] Chung K.-R. Wu P.-C. Chen Y.-K. Yago J. I. (2020). The SreA repressor required for growth and suppression of siderophore biosynthesis, hydrogen peroxide resistance, cell wall integrity, and virulence in the phytopathogenic fungus *Alternaria alternata*. Fungal Genet. Biol. 139:103384. 10.1016/j.fgb.2020.103384 32278718

[ref14] Dammai V. Subramani S. (2001). The human peroxisomal targeting signal receptor, Pex5p, is translocated into the peroxisomal matrix and recycled to the cytosol. Cell 105, 187–196. 10.1016/S0092-8674(01)00310-5 11336669

[ref15] Farré J.-C. Mahalingam S. S. Proietto M. Subramani S. (2019). Peroxisome biogenesis, membrane contact sites, and quality control. EMBO Rep. 20:e46864. 10.15252/embr.201846864 30530632 PMC6322382

[ref16] Farré J.-C. Subramani S. (2016). Mechanistic insights into selective autophagy pathways: lessons from yeast. Nat. Rev. Mol. Cell Biol. 17, 537–552. 10.1038/nrm.2016.74 27381245 PMC5549613

[ref17] Freitag J. Ast J. Linne U. Stehlik T. Martorana D. Bölker M.. (2014). Peroxisomes contribute to biosynthesis of extracellular glycolipids in fungi. Mol. Microbiol. 93, 24–36. 10.1111/mmi.12642 24835306

[ref18] Fu H. Chung K.-R. Li H. (2020). The *Alternaria alternata* basal transcription factor II H subunit tfb5 required for DNA repair, toxin production, stress resistance and pathogenicity in citrus. Mol. Plant Pathol. 21, 1337–1352. 10.1111/mpp.12982 32776683 PMC7488464

[ref19] Fujihara N. Sakaguchi A. Tanaka S. Fujii S. Tsuji G. Shiraishi T.. (2010). Peroxisome biogenesis factor PEX13 is required for appressorium-mediated plant infection by the anthracnose fungus *Colletotrichum orbiculare*. Mol. Plant Microbe. Interact. 23, 436–445. 10.1094/MPMI-23-4-0436 20192831

[ref20] Germain K. Kim P. K. (2020). Pexophagy: a model for selective autophagy. Intl. J. Mol. Sci. 21:578. 10.3390/ijms21020578 PMC7013971 31963200

[ref21] Goh J. Jeon J. Kim K. S. Park J. Park S.-Y. Lee Y.-H. (2011). The pex7-mediated peroxisomal import system is required for fungal development and pathogenicity in *Magnaporthe oryzae*. PLoS One 6:e28220. 10.1371/journal.pone.0028220 22194815 PMC3237427

[ref22] Gould S. J. Collins C. S. (2002). Peroxisomal-protein import: is it really that complex? Nat. Rev. Mol. Cell Biol. 3, 382–389. 10.1038/nrm807 11988772

[ref23] Gould S. J. Valle D. (2000). Peroxisome biogenesis disorder. Trend. Genet. 16, 340–345. 10.1016/j.bbamcr.2006.09.010 10904262

[ref24] Gow N. A. R. Latge J.-P. Munro C. A. (2017). The fungal cell wall: structure, biosynthesis, and function. Microbiol. Spectr. 5:FUNK-0035-2016. 10.1128/microbiolspec.FUNK-0035-2016 PMC11687499 28513415

[ref25] Gründlinger M. Yasmin S. Lechner B. E. Geley S. Schrettl M. Hynes M.. (2013). Fungal siderophore biosynthesis is partially localized in peroxisomes. Mol. Microbiol. 88, 862–875. 10.1111/mmi.12225 23617799 PMC3709128

[ref26] Heiland I. Erdmann R. (2005). Biogenesis of peroxisomes topogenesis of the peroxisomal membrane and matrix proteins. FEBS J. 272, 2362–2372. 10.1111/j.1742-4658.2005.04690.x 15885087

[ref27] Holroyd C. Erdmann R. (2001). Protein translocation machineries of peroxisomes. FEBS Lett. 501, 6–10. 10.1016/S0014-5793(01)02617-5 11457447

[ref28] Hu J. Baker A. Bartel B. Linka N. Mullen R. T. Reumann S.. (2012). Plant peroxisomes: biogenesis and function. Plant Cell 24, 2279–2303. 10.1105/tpc.112.096586 22669882 PMC3406917

[ref29] Hutchins M. U. Marten Veenhuis M. Klionsky D. J. (1999). Peroxisome degradation in *Saccharomyces cerevisiae* is dependent on machinery of macroautophagy and the Cvt pathway. J. Cell Sci. 112, 4079–4087. 10547367 10.1242/jcs.112.22.4079

[ref30] Idnurm A. Giles S. S. Perfect J. R. Heitman J. (2007). Peroxisome function regulates growth on glucose in the basidiomycete *Cryptococcus neoformans*. Eukaryot. Cell 6, 60–72. 10.1128/EC.00214-06, PMID: 17041184 PMC1800366

[ref31] Imazaki A. Tanaka A. Harimoto Y. Yamamoto M. Akimitsu K. Park P.. (2010). Contribution of peroxisomes to secondary metabolism and pathogenicity in the fungal plant pathogen *Alternaria alternata*. Eukaryot. Cell 9, 682–694. 10.1128/EC.00369-09, PMID: 20348386 PMC2863954

[ref32] Kimura A. Takano Y. Furusawa I. Okuno T. (2001). Peroxisomal metabolic function is required for appressorium-mediated plant infection by *Colletotrichum lagenarium*. Plant Cell 13, 1945–1957. 10.1105/tpc.010084, PMID: 11487704 PMC139132

[ref33] Kohmoto K. Itoh Y. Shimomura N. Kondoh Y. Otani H. Nishimura S.. (1993). Isolation and biological activities of two host-specific toxins from tangerine pathotype of *Alternaria alternata*. Phytopathology 83, 495–502.

[ref34] Law K. B. Tinkew D. B. Pietro E. D. Snowden A. Jones R. O. Moser A.. (2017). The peroxisomal AAA ATPase complex prevents pexophagy and development of peroxisome biogenesis disorders. Autophagy 13, 868–884. 10.1080/15548627.2017.1291470, PMID: 28521612 PMC5446072

[ref35] Li L. Wang J. Zhang Z. Wang Y. Liu M. Jiang H.. (2014). Mopex19, which is essential for maintenance of peroxisomal structure and Woronin bodies, is required for metabolism and development in the rice blast fungus. PLoS One 9:e85252. 10.1371/journal.pone.0085252 24454828 PMC3891873

[ref36] Lin C.-H. Chung K.-R. (2010). Specialized and shared functions of the histidine kinase- and HOG1 MAP kinase-mediated signaling pathways in *Alternaria alternata*, a filamentous fungal pathogen of citrus. Fungal Genet. Biol. 47, 818–827. 10.1016/j.fgb.2010.06.009, PMID: 20601043

[ref37] Lin C.-H. Yang S. L. Chung K.-R. (2009). The YAP1 homolog-mediated oxidative stress tolerance is crucial for pathogenicity of the necrotrophic fungus *Alternaria alternata* in citrus. Mol. Plant Microbe Interact. 22, 942–952. 10.1094/MPMI-22-8-0942, PMID: 19589070

[ref38] Lin C.-H. Yang S. L. Chung K.-R. (2011). Cellular responses required for oxidative stress tolerance, colonization and lesion formation by the necrotrophic fungus *Alternaria alternata* in citrus. Curr. Microbiol. 62, 807–815. 10.1007/s00284-010-9795-y, PMID: 20978890

[ref39] Lin H. -C. Yu P.-L. Chen L.-H. Tsai H.-C. Chung K.-R. (2018). A major facilitator superfamily transporter regulated by the stress-responsive transcription factor Yap1 is required for resistance to fungicides, xenobiotics, and oxidants and full virulence in *Alternaria alternata*. Front. Microbiol. 9:2229. 10.3389/fmicb.2018.02229 30279684 PMC6153361

[ref40] Lipka V. Dittgen J. Bednarek P. Bhat R. Wiermer M. Stein M.. (2005). Pre- and postinvasion defenses both contribute to nonhost resistance in *Arabidopsi*s. Science 310, 1180–1183. 10.1126/science.1119409, PMID: 16293760

[ref41] Ma H. Wang M. Gai Y. Fu H. Ruan R. Chung K.-R.. (2018). Thioredoxin and glutaredoxin systems required for oxidative stress resistance, fungicide sensitivity and virulence of *Alternaria alternata*. Appl. Environ. Microbiol. 84:e00086-18. 10.1128/AEM.00086-18 29752269 PMC6029089

[ref42] Magliano P. Flipphi M. Arpat B. A. Delessert S. Poirier Y. (2011). Contributions of the peroxisome and β-oxidation cycle to biotin synthesis in fungi. J. Biol. Chem. 286, 42133–42140. 10.1074/jbc.M111.279687 21998305 PMC3234907

[ref43] Mahalingam S. S. Shukla N. Farré J.-C. Zientara-Rytter K. Subramani S. (2021). Balancing the opposing principles that govern peroxisome homeostasis. Trends Biochem. Sci. 46, 200–212. 10.1016/j.tibs.2020.09.006, PMID: 33046344 PMC7880872

[ref44] Manjithaya R. Jain S. Farré J. C. Subramani S. (2010b). A yeast MAPK cascade regulates pexophagy but not other autophagy pathways. J. Cell Biol. 189, 303–310. 10.1083/jcb.200909154, PMID: 20385774 PMC2856896

[ref45] Manjithaya R. Nazarko T. Y. Farré J.-C. Subramani S. (2010a). Molecular mechanism and physiological role of pexophagy. FEBS Lett. 584, 1367–1373. 10.1016/j.febslet.2010.01.019, PMID: 20083110 PMC2843806

[ref46] Maruyama J.-I. Kitamoto K. (2013). Expanding functional repertoires of fungal peroxisomes: contribution to growth and survival processes. Front. Physiol. 4:177. 10.3389/fphys.2013.00177 23882222 PMC3713238

[ref47] Maruyama J.-I. Yamaoka S. Matsuo I. Tsutsumi N. Kitamoto K. (2012). A newly discovered function of peroxisomes: involvement in biotin biosynthesis. Plant Signal. Behav. 7, 1589–1593. 10.4161/psb.22405, PMID: 23073000 PMC3578898

[ref48] Min K. Son H. Lee J. Choi G. J. Kim J.-C. Lee Y.-W. (2012). Peroxisome function is required for virulence and survival of *Fusarium graminearum*. Mol. Plant-Microbe Interact. 25, 1617–1627. 10.1094/MPMI-06-12-0149-R 22913493

[ref49] Nuttall J. M. Motley A. M. Hettema E. H. (2014). Deficiency of the exportomer components Pex1, Pex6, and Pex15 causes enhanced pexophagy in *Saccharomyces cerevisiae*. Autophagy 10, 835–845. 10.4161/auto.28259, PMID: 24657987 PMC5119063

[ref50] Oku M. Sakai Y. (2010). Peroxisomes as dynamic organelles: autophagic degradation. FEBS J. 277, 3289–3294. 10.1111/j.1742-4658.2010.07741.x, PMID: 20629742

[ref51] Perry R. J. Mast F. D. Rachubinski R. A. (2009). Endoplasmic reticulum-associated secretory proteins Sec20p, Sec39p, and Dsl1p are involved in peroxisome biogenesis. Eukaryot. Cell 8, 830–843. 10.1128/EC.00024-09, PMID: 19346454 PMC2698310

[ref52] Platta H. W. Erdmann R. (2007a). Peroxisomal dynamics. Trend. Cell Biol. 17, 474–484. 10.1016/j.tcb.2007.06.009 17913497

[ref53] Platta H. W. Erdmann R. (2007b). The peroxisomal protein import machinery. FEBS Lett. 581, 2811–2819. 10.1016/j.febslet.2007.04.001 17445803

[ref54] Rucktäschel R. Girzalsky W. Erdmann R. (2011). Protein import machineries of peroxisomes. Biochim. Biophys. Acta 1808, 892–900. 10.1016/j.bbamem.2010.07.020, PMID: 20659419

[ref55] Sakai Y. Oku M. van der Klei I. J. Kiel J. A. K. W. (2006). Pexophagy: autophagic degradation of peroxisomes. Biochim. Biophys. Acta 1763, 1767–1775. 10.1016/j.bbamcr.2006.08.023, PMID: 17005271

[ref56] Schefe J. H. Lehmann K. E. Buschmann I. R. Unger T. Funke-Kaiser H. (2006). Quantitative real-time RT-PCR data analysis: current concepts and the novel “gene expression’s CT difference” formula. J. Mol. Med. 84, 901–910. 10.1007/s00109-006-0097-6 16972087

[ref57] Schrader M. Fahimi H. D. (2006). Peroxisomes and oxidative stress. Biochim. Biophys. Acta 1763, 1755–1766. 10.1016/j.bbamcr.2006.09.006, PMID: 17034877

[ref58] Schwyn B. Neilands J. B. (1987). Universal chemical assay for the detection and determination of siderophores. Anal. Biochem. 160, 47–56. 10.1016/0003-2697(87)90612-9, PMID: 2952030

[ref59] Selvaggini S. Munro C. A. Paschoud S. Sanglard D. Gow N. A. R. (2017). Independent regulation of chitin synthase and chitinase activity in *Candida albicans* and *Saccharomyces cerevisiae*. Microbiology 150, 921–928. 10.1099/mic.0.26661-0, PMID: 15073301

[ref60] Sengupta S. Chowdhury S. Bose Dasgupta S. Wright C. W. Majumder H. (2011). Cryptolepine-induced cell death of *Leishmania donovani* promastigotes is augmented by inhibition of autophagy. Mol. Biol. Int. 2011:187850. 10.4061/2011/187850, PMID: 22091398 PMC3195846

[ref61] Sibirny A. A. (2016). Yeast peroxisomes: structure, functions and biotechnological opportunities. FEMS Yeast Res. 16:fow038. 10.1093/femsyr/fow038 27189367

[ref62] Smith J. J. Aitchison J. D. (2009). Regulation of peroxisome dynamics. Curr. Opin. Cell Biol. 21, 119–126. 10.1016/j.ceb.2009.01.009, PMID: 19188056 PMC2681484

[ref63] Stehlik T. Sandrock B. Ast J. Freitag J. (2014). Fungal peroxisomes as biosynthetic organelles. Curr. Opin. Microbiol. 22, 8–14. 10.1016/j.mib.2014.09.011, PMID: 25305532

[ref64] Sun G. Elowsky C. Li G. Wilson R. A. (2018). TOR-autophagy branch signaling via Imp1 dictates plant-microbe biotrophic interface longevity. PLoS Genet. 15:e1008016. 10.1371/journal.pgen.1008016 PMC6281275 30462633

[ref65] Sweigard J. A. Chumley F. C. Carroll A. M. Farrall L. Valent B. (1997). A series of vectors for fungal transformation. Fungal Genet. Newsl. 44, 52–53. 10.4148/1941-4765.1287

[ref66] Tabak H. F. van der Zand A. Braakman I. (2008). Peroxisomes: minted by the ER. Curr. Opin. Cell Biol. 20, 393–400. 10.1016/j.ceb.2008.05.008, PMID: 18619829

[ref67] Tanabe Y. Maruyama J. I. Yamaoka S. Yahagi D. Matsuo I. Tsutsumi N.. (2011). Peroxisomes are involved in biotin biosynthesis in *Aspergillus* and *Arabidopsis*. J. Biol. Chem. 286, 30455–30461. 10.1074/jbc.M111.247338 21730067 PMC3162405

[ref68] Titorenko V. I. Rachubinski R. A. (1998). Mutants of the yeast *Yarrowia lipolytica* defective in protein exit from the endoplasmic reticulum are also defective in peroxisome biogenesis. Mol. Cell. Biol. 18, 2789–2803. 10.1128/mcb.18.5.2789, PMID: 9566898 PMC110658

[ref69] Titorenko V. I. Rachubinski R. A. (2011). Dynamics of peroxisome assembly and function. Trend. Cell. Biol. 11, 22–29. 10.1016/S0962-8924(00)01865-1 11146295

[ref70] Wanders R. J. Ferdinandusse S. Brites P. Kemp S. (2010). Peroxisomes, lipid metabolism and lipotoxicity. Biochim. Biophys. Acta 1801, 272–280. 10.1016/j.bbalip.2010.01.001, PMID: 20064629

[ref71] Wang P.-H. Wu P.-C. Huang R. Chung K.-R. (2020). The role of a nascent polypeptide-associated complex subunit alpha in siderophore biosynthesis, oxidative stress response and virulence in *Alternaria alternata*. Mol. Plant-Microbe Interact. 33, 668–679. 10.1094/MPMI-11-19-0315-R 31928525

[ref72] Wang Z.-Y. Soanes D. M. Kershaw M. J. Talbot N. J. (2007). Functional analysis of lipid metabolism in *Magnaporthe grisea* reveals a requirement for peroxisomal fatty acid β-oxidation during appressorium-mediated plant infection. Mol. Plant-Microbe Interact. 20, 475–491. 10.1094/MPMI-20-5-0475, PMID: 17506326

[ref73] Watanabe T. Koller K. Messner K. (1998). Copper-dependent depolymerization of lignin in the presence of fungal metabolite, pyridine. J. Biotechnol. 62, 221–230. 10.1016/s0168-1656(98)00063-7, PMID: 9729805

[ref74] Wu P.-C. Chen C.-W. Choo C. Y. L. Chen Y.-K. Yago J. I. Chung K.-R. (2020a). Proper functions of peroxisomes are vital for pathogenesis of citrus brown spot disease caused by *Alternaria alternata*. J. Fungi 6:248. 10.3390/jof6040248 PMC7712655 33114679

[ref75] Wu P.-C. Chen C.-W. Choo C. Y. L. Chen Y.-K. Yago J. I. Chung K.-R. (2020b). Biotin biosynthesis regulated by NADPH oxidases and lipid metabolism is required for infectivity in the citrus fungal pathogen *Alternaria alternata*. Microbiol. Res. 241:126566. 10.1016/j.micres.2020.126566 33032167

[ref76] Xu J.-R. (2000). MAP kinases in fungal pathogens. Fungal Genet. Biol. 31, 137–152. 10.1006/fgbi.2000.1237 11273677

[ref77] Yago J. I. Lin C.-H. Chung K.-R. (2011). The SLT2 MAP kinase-mediated signalling pathway governs conidiation, morphogenesis, fungal virulence, and production of toxin and melanin in the tangerine pathotype of *Alternaria alternata*. Mol. Plant Pathol. 12, 653–665. 10.1111/j.1364-3703.2010.00701.x, PMID: 21726368 PMC6640243

[ref78] Yang S. L. Chung K.-R. (2012). The NADPH oxidase-mediated production of H_2_O_2_ and resistance to oxidative stress in the necrotrophic pathogen *Alternaria alternata* of citrus. Mol. Plant Pathol. 13, 900–914. 10.1111/j.1364-3703.2012.00799.x, PMID: 22435666 PMC6638813

[ref79] Yang S. L. Chung K.-R. (2013). Similar and distinct roles of NADPH oxidase components in the tangerine pathotype of *Alternaria alternata*. Mol. Plant Pathol. 14, 543–556. 10.1111/mpp.12026, PMID: 23527595 PMC6638896

[ref80] Yang S. L. Lin C. H. Chung K. R. (2009). Coordinate control of oxidative stress, vegetative growth and fungal pathogenicity via the AP1-mediated pathway in the rough lemon pathotype of *Alternaria alternata*. Physiol. Mol. Plant Pathol. 74, 100–110. 10.1016/j.pmpp.2009.09.007

[ref81] Yang S. L. Yu P.-L. Chung K.-R. (2016). The glutathione peroxidase–mediated reactive oxygen species resistance, fungicide sensitivity and cell wall construction in the citrus fungal pathogen *Alternaria alternata*. Environ. Microbiol. 18, 923–935. 10.1111/1462-2920.13125, PMID: 26567914

[ref82] Yu P.-L. Chen L.-H. Chung K.-R. (2016). How the pathogenic fungus *Alternaria alternata* copes with stress via the response regulators SSK1 and SHO1. PLoS One 11:e0149153. 10.1371/journal.pone.0149153 26863027 PMC4749125

[ref83] Zhang L. Wang L. Liang Y. Yu J. (2019). FgPEX4 is involved in development, pathogenicity, and cell wall integrity in *Fusarium graminearum*. Curr. Genet. 65, 747–758. 10.1007/s00294-018-0925-6, PMID: 30603875

