# Supplementary material for: Peroxisomes Implicated in the Biosynthesis of Siderophores and Biotin, Cell Wall Integrity, Autophagy, and Response to Hydrogen Peroxide in the Citrus Pathogenic Fungus Alternaria alternata
Source: Front Microbiol. 2021 Jun 28;12:645792. doi: 10.3389/fmicb.2021.645792 (PMC8273606; doi:10.3389/fmicb.2021.645792)
Supplement: Supplementary file 1 [file Data_Sheet_1.docx]

Supplementary Material

**Supplementary Table S1. Oligonucleotide primers used in this study**

| **Primer** | **Sequence (5’→3’)** | **Remarks** |
| --- | --- | --- |
| trpCP-R-plus mcherry | tcgcccttgctcaccatatgcttgggtagaataggtaagtcagattg | *trpC* promoter overlapping with mCherry (reverse) |
| mCherry-SKL-R | atggagctattaaatcattatagcttcgacttgtacagctcgtccatgcc | mCherry gene with SKL (reverse) |
| mCherry-F-New | atggtgagcaagggcgagga | mCherry gene (forward) |
| mCherry-R | atggagctattaaatcattacttgtacagctcgtccatgcc | mCherry gene (reverse) |
| De-SKL-F | gcatggacgagctgtacaagtaatgatttaatagctccatg | *trpC* terminator overlapping with SKL (forward) |
| trpCP-F-BamHI | actaggatcccagaagatgatattgaaggagcatttttggg | *trpC* promoter (forward) |
| trpCT-F | tgatttaatagctccatgtcaacaagaataaaacgc | *trpC* terminator (forward) |
| trpCT-R-HindIII | tagcaagcttaaagaaggattacctctaaacaagtgtacctg | *trpC* terminator (reverse) |


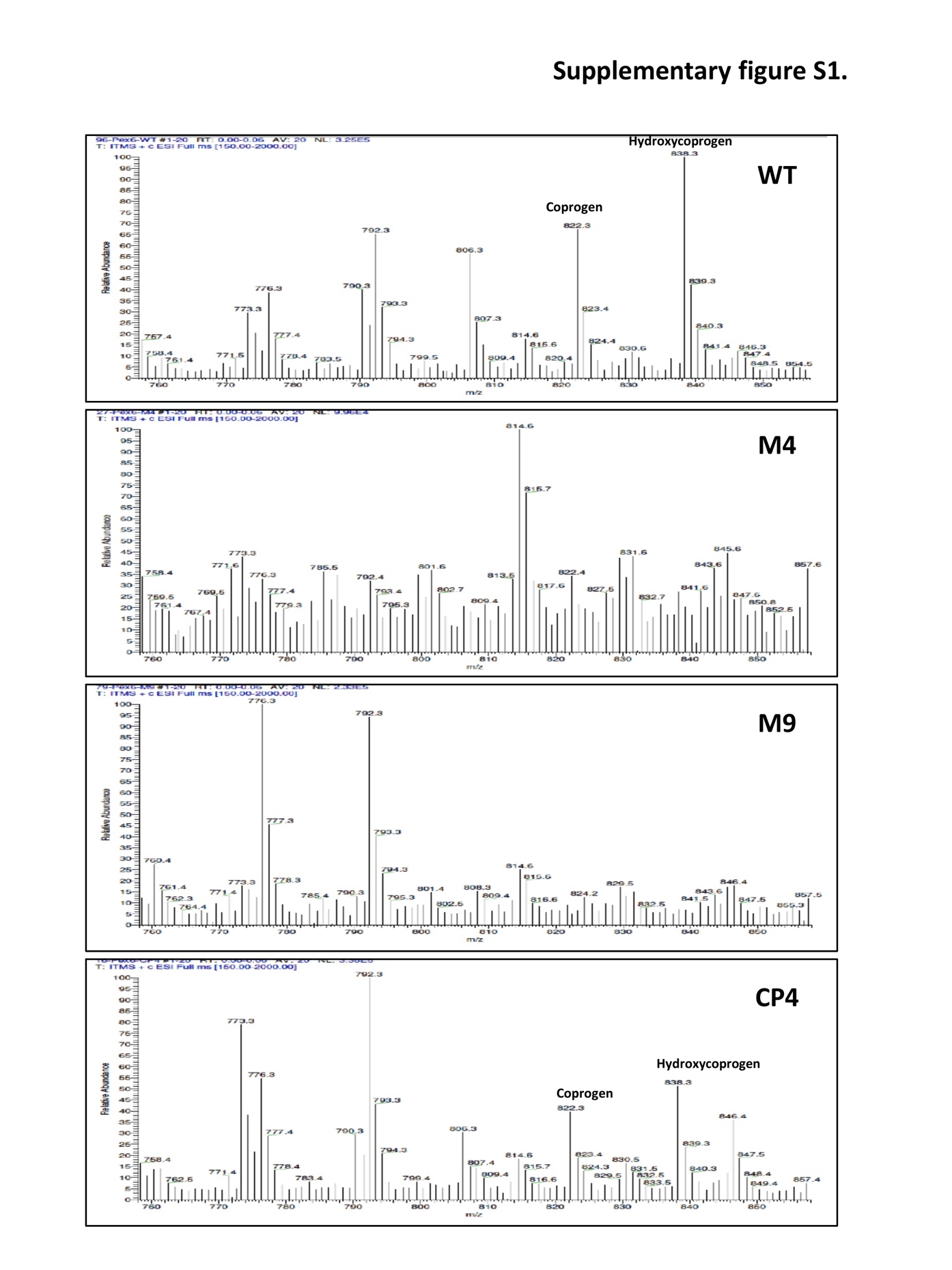


**Supplementary Figure S1** Confirmation of siderophores produced by wild-type, the Δ*pex6* mutants (M4 and M9) and CP4 strains by liquid chromatography-tandem mass spectrometry (LC/MS/MS).
